# Soft‐Actuated Cuff Electrodes with Minimal Contact for Bidirectional Peripheral Interfaces

**DOI:** 10.1002/adma.202409942

**Published:** 2024-11-10

**Authors:** Hyunmin Moon, Byungwook Park, Namsun Chou, Ki‐Su Park, Sanghoon Lee, Sohee Kim

**Affiliations:** ^1^ Department of Robotics and Mechatronics Engineering Daegu Gyeongbuk Institute of Science and Technology (DGIST) Daegu 42988 Republic of Korea; ^2^ Department of Mechanical Engineering Massachusetts Institute of Technology Cambridge MA 02139 USA; ^3^ Emotion, Cognition, and Behavior Research Group Korea Brain Research Institute Daegu 41062 Republic of Korea; ^4^ Department of Neurosurgery Kyungpook National University School of Medicine Daegu 41944 Republic of Korea

**Keywords:** bidirectional interface, fluid injection, minimal contact, soft actuation, soft‐actuated cuff electrodes (SACE), sutureless cuff electrode

## Abstract

Neural interfaces with embedded electrical functions, such as cuff electrodes, are essential for monitoring and stimulating peripheral nerves. Still, several challenges remain with cuff electrodes because sutured devices can damage the nerve by high pressure and the secured contact of electrodes with the nerve is hard to accomplish, which however is essential in maintaining electrical performance. Here, a sutureless soft‐actuated cuff electrodes (SACE) that can envelop the nerve conveniently by creating a bent shape controlled upon fluid injection, is introduced. Moreover, fluid injection protrudes part of the device where electrodes are formed, thereby achieving minimized, soft but secure contact between the electrodes and the nerve. In vivo results demonstrate the successful recording and stimulation of peripheral nerves over time up to 6 weeks. While securing contact with the nerve, the implanted electrodes can preserve the nerve intact with no reduction in blood flow, thereby indicating only minimal compressive force applied to the nerve. The SACE is expected to be a promising tool for recording and stimulation of peripheral nerves toward bidirectional neuroprostheses.

## Graphical abstract

Soft‐actuated cuff electrodes (SACE) for bidirectional peripheral interfaces enable minimal and secure contact to the nerve through fluid injection‐based soft actuation. A 3D bent structure can grasp and securely contact the nerves with only a little pressure (<1.21 gram‐force). The SACE can achieve negligible damage to the nerve during recording of sensory and motor feedback signals with superior SNR and neuromodulation for long‐term studies.

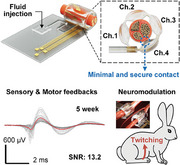

## 1 Introduction

Neuroprostheses for neuromodulation and neural signal recording have contributed to the functional restoration of patients with neurological disorders such as tetraplegia, stroke, depression, epilepsy, and degenerative diseases.^[^ ^1^, ^2^, ^3^, ^4^, ^5^, ^6^, ^7^, ^8^ ^]^ Electrodes to communicate with the nervous systems are essential to the clinical purpose of neuroprostheses. In particular, soft and flexible electrodes composed of polymeric substrates have been developed for use in the peripheral nervous system (PNS) due to the limitations originating from those made of inorganic materials such as silicon.^[^ ^9^, ^10^, ^11^, ^12^, ^13^ ^]^ To achieve ideal neural interfaces for the PNS, the bioelectronics such as cuff electrodes need to meet several requirements: biocompatibility, the softness of substrate materials^[^ ^14^, ^15^, ^16^ ^]^ and convenient implantation with minimal nerve damage.^[^ ^16^, ^17^, ^18^ ^]^

The prognosis after the implantation of neuroprostheses significantly depends on the biocompatibility and softness of the substrate materials used.^[^ ^15^, ^19^ ^]^ Though various cuff electrodes based on polyimide or parylene C have been fabricated,^[^ ^17^, ^20^, ^21^, ^22^ ^]^ Young's moduli of these polymers (polyimide: ≈2.5 GPa; parylene C: ≈2.76 GPa) remain >4700 times larger than that of neural tissues (rat sciatic nerve: ≈580 kPa).^[^ ^23^ ^]^ This mechanical mismatch between the substrate and the peripheral nerve can cause nerve damage, such as scarring, inflammation, and foreign body response.^[^ ^14^, ^16^ ^]^ Though thin films of these substrates under several microns can compensate the mechanical mismatch to the peripheral nerves,^[^ ^24^, ^25^ ^]^ chronic studies have been challenging because the intimate interfaces cannot be controlled. For example, during implantation, cuff electrodes tightly envelop the nerve using a surgical string, causing nerve damage by local compression.^[^ ^26^, ^27^, ^28^ ^]^ Moreover, suturing cuff electrodes around the nerve is a specialized and complicated step, which significantly affects recording or stimulation success.^[^ ^21^, ^29^ ^]^ Therefore, several mechanisms have been developed to conveniently implant cuff electrodes, such as self‐sizing,^[^ ^30^ ^]^ self‐closing,^[^ ^31^, ^32^ ^]^ self‐locking,^[^ ^21^ ^]^ C‐shaped,^[^ ^33^ ^]^ clip,^[^ ^29^ ^]^ and climbing‐inspired twining electrodes.^[^ ^16^ ^]^ These previous studies reported successful stimulation or recording of peripheral nerves. However, the main challenge of such devices is to guarantee intimate contact between the electrodes and nerves, enabling minimal nerve damage and secure communication with the nerves.^[^ ^17^, ^20^, ^21^, ^29^, ^34^, ^35^ ^]^ To be specific, high compression by the implanted cuff electrodes can cause nerve damage, while weak contact between the electrodes and the nerve induces lower electrical performance and movement of the electrodes relative to the nerve. These problems from high compression or weak contact eventually deteriorate the stimulation or signal recording performance.

In this study, we propose a sutureless, soft‐actuated cuff electrodes (SACE) device that grasps peripheral nerves by itself upon fluid injection into part of the device made of soft and expandable polymeric structures. In addition, 3D convex structures embedded in the developed device facilitate the minimal and secure contact between the electrodes and target nerves. Upon fluid injection, the 2D planar device turns into a 3D bent structure that can grasp and securely contact the nerves, resulting low noise level and high signal‐to‐noise ratio (SNR) in signal recording. Soft polydimethylsiloxane (PDMS) is used as the base material of the developed device, which has the highest biocompatibility of US Pharmacopeia Class VI and an elastic modulus of 360–868 kPa,^[^ ^36^ ^]^ similar to that of nerves (≈580 kPa). The PDMS‐based electrodes are expected to reduce nerve damage from the mechanical mismatch between the electrodes and the nerve, compared to polyimide‐ or parylene C‐based cuff electrodes. In addition, the entire fabrication of the electrodes employs MEMS techniques on a single wafer, enabling mass production. We demonstrate the developed device in long‐term recording and stimulation of in vivo peripheral nerves for up to 6 weeks. Both sensory and motor feedback signals of compound nerve action potentials (CNAPs) can be acquired in response to various stimuli, i.e., electrical nerve stimulation, tactile stimulation, and involuntary forced leg movements. In addition, selective nerve stimulation at six different regions is demonstrated by detecting electromyograms (EMGs) from gastrocnemius (GN) and tibialis anterior (TA) muscles. We also confirm that the implantation of the developed SACE device did not damage the nerve by compression, through the histological examination of morphology and cell functions as well as blood flow measurement.

## 2 Results

### 2.1 SACE with Minimal and Secure Contact

**Figure 1:**
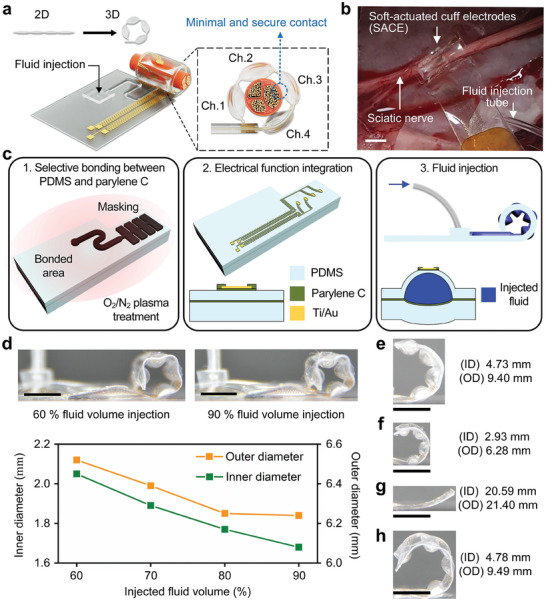
Soft‐actuated cuff electrodes (SACE) to communicate with nerves. a) Conceptual illustration of the SACE device: a flat device turns into a 3D bent structure by inflating balloons via fluid injection. The inflated balloons carry four electrode channels and securely grasp the nerve with minimal contact. b) Image of a rabbit sciatic nerve enveloped by the SACE. The scale bar is 5 mm. c) Schematic illustration of the device design and architecture that comprise the selectively bonded substrate (PDMS‐parylene C‐PDMS) including electrode patterns. Fluid injection generates balloon inflation for minimal and secure contact with a peripheral nerve. d) Images of the bent SACE with optimized balloon dimensions: 100 µm of top thickness, 200 µm of bottom thickness, 3 mm of balloon length, and 1 mm of balloon gap. The ID and OD of the bent device after injection with 60% to 90% of the maximum fluid volume are plotted. For (e–h), injection with 90% of the maximum fluid volume is used to bend the devices. e) The top and bottom PDMS layers thicker than the optimized dimensions (200 and 400 µm, respectively) reduce the degree of bending. The ID and OD of the bent shape are larger when the balloon length is smaller, indicating that a smaller balloon causes less bending: balloon lengths of f) 2 mm and g) 1 mm, respectively. A larger gap between balloons increases the bending diameter, as shown in the device h) with a 2 mm gap compared to the device d) with a 1 mm gap. The scale bars are 5 mm.

**Figure** **1**a shows the schematic of the SACE that envelops a nerve upon fluid injection. The array of inflated balloons can securely grab the nerve by forming a bent shape with minimal and secure contact to the nerve. The SACE was applied to an animal's sciatic nerve for neural signal recording or neuromodulation (Figure 1b). The developed device was composed of a soft and flexible substrate made of top PDMS, middle parylene C, and bottom PDMS layers (Figure 1c). The device was fabricated by selectively bonding the top PDMS and middle parylene C layers by plasma treatment, generating a balloon pattern.^[^ ^37^ ^]^ Metal electrodes were deposited on the top PDMS layer to add electrical functionalities, followed by parylene C insulation, and fluid injection generated 3D structure underneath the electrodes. The details of the fabrication processes are described in Experimental Section and Figure S1 (Supporting Information). Both the inner (ID) and outer (OD) diameters of the bent device could be precisely controlled by the injected fluid volume (Figure 1d). Precise control of the bending diameter was essential to realize the cuff electrodes with secure contact with target nerves. A larger amount of injected fluid forced the developed device to bend more: the ID and OD of the bent shape changed from 2.05 to 1.68 mm and 6.52 to 6.24 mm, respectively, when 60% to 90% of the maximum injectable fluid volume was injected. The maximum injectable fluid volume before the device burst was measured experimentally, and subsequent experiments used 90% of the maximum injectable fluid volume (Figure S2, Supporting Information).

The injected fluid volume, the relative thicknesses of the top and bottom PDMS layers, and the dimensions of the balloons were critical for controlling the diameter of the bent shape (Figure S3, Supporting Information). Six representative samples were fabricated with different combinations of four parameters: top PDMS thickness; bottom PDMS thickness; balloon length; and balloon gap (Table S1, Supporting Information). Figures 1e–h and S4a,b (Supporting Information) show each sample's measured ID and OD after injection with 90% of the maximum fluid volume. No bent shape was obtained when the thicknesses of the top and bottom PDMS layers were the same. However, the device was bent when the bottom PDMS layer was thicker than the top PDMS layer. The bent shape's ID and OD were larger with thicker PDMS layers, shorter balloon length, and longer balloon gap. By controlling these parameters, various IDs of the bent shape, ranging from 1.68 to 20.59 mm, were achieved. In particular, given that the rabbit sciatic nerve has a minimum diameter of 2.2 mm, only the device with a 100 µm thick top PDMS layer, 200 µm thick bottom PDMS layer, 3 mm long balloon length, and 1 mm long gap between balloons had an ID < 2.2 mm (1.68 mm). This device was hereafter used for all experiments since it could guarantee secure contact between the electrodes and the rabbit's sciatic nerve. Furthermore, the developed device with four balloons could form contacts to nerve models with diameters ranging from 1.5 to 10 mm after injection with 90% of the maximum fluid volume (Figure S5, Supporting Information). The resulted contact force was measured to be smaller than 1.21 gram–force (gf), expecting minimal nerve damage when the developed device is implanted (Figure S6, Supporting Information). The contact force tended to slightly increase with large nerve diameter, ranging from 0.8 to 0.96 g for 2–3 mm diameters. In addition, the developed device with four balloons could fully wrap around the nerves with diameters ranging from 1.68 to 3 mm, which includes sciatic nerves enlarged by the growth of the animal. The number of balloons needs to be increased to completely wrap a larger nerve: for example, the device with five balloons could fully wrap around the canine sciatic nerve of 3.5 mm diameter.

### 2.2 Electrochemical Characterization: Reusability, Long‐Term Stability, and Repeatability

To validate the electrical functionality of the device, we analyzed the electrochemical impedance and charge storage capacity (CSC) after injection of 0%, 60%, and 90% of the maximum fluid volume (Figure S7, Supporting Information). The impedance magnitude increased after fluid injection, but did not exceed 1.15 kΩ at 1 kHz as the injected volume increased. This increase in impedance was speculated to originate from microcracks in the stretched metal patterns caused by fluid injection. In addition, the cyclic voltammetry (CV) curves show that the CSC increased slightly from 0.28 to 0.32 mC cm^−2^ after fluid injection. This increase in CSC could be explained by the increased surface area caused by metal pattern stretching.

**Figure 2:**
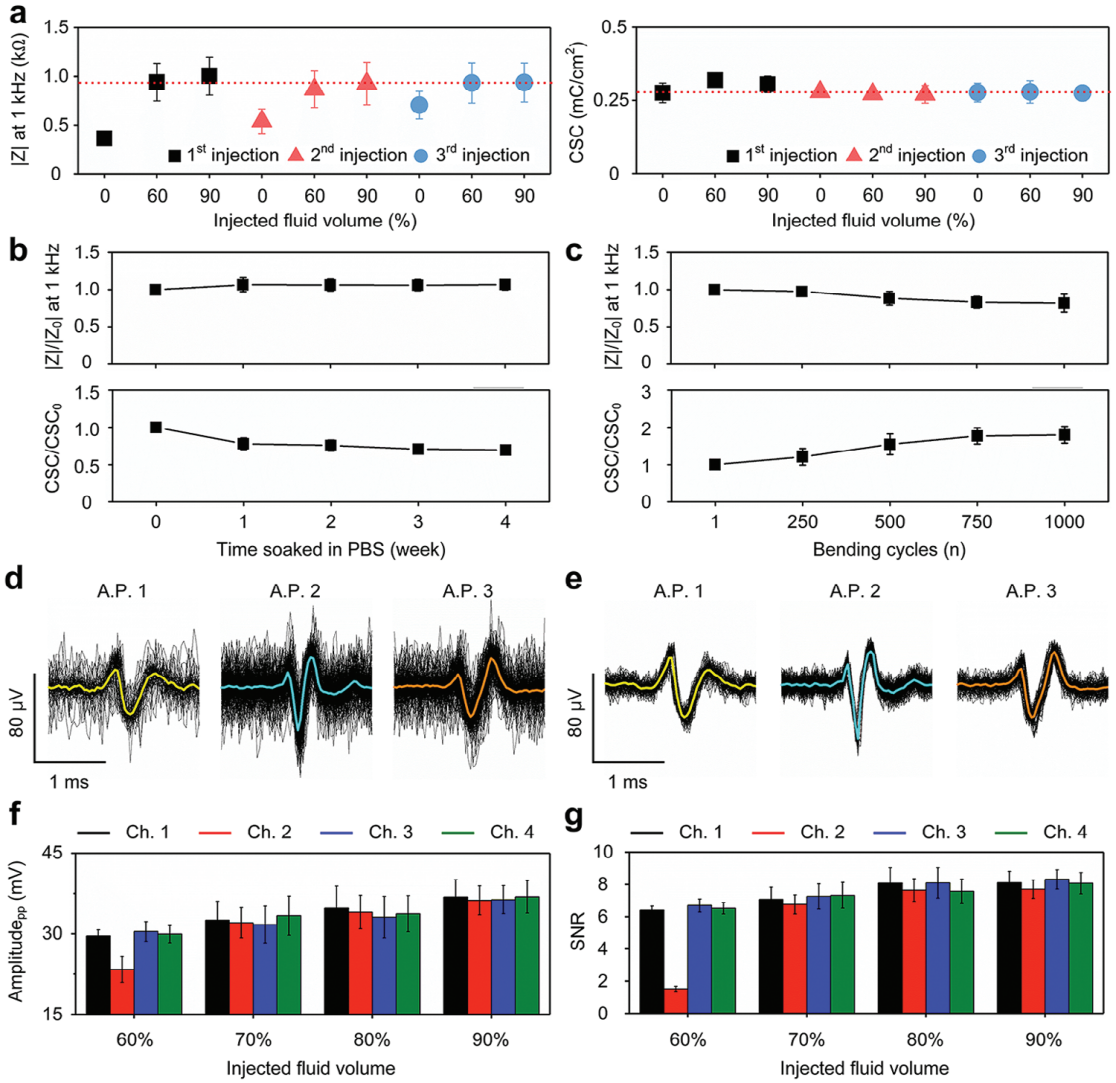
Characterizations of electrodes for electrochemical properties and AP recordings upon fluid injection. a) Reusability: impedance at 1 kHz and CSC measured after the first, second, and third injections with 0%, 60%, and 90% of the maximum fluid volume (*n* ≥ 9 for each impedance and CSC measurement). The red‐dashed lines indicate the convergence of the impedance and CSC after the third injection. b) Long‐term stability: changes in impedance at 1 kHz and CSC over time for a device soaked in PBS for up to 4 weeks (*n* ≥ 12). c) Repeatability: changes in impedance at 1 kHz and CSC under repeated bending up to 1000 cycles (*n* ≥ 12). d,e) Overlapped AP waveforms (black line) and their averages (yellow, cyan, and orange lines) after injection with d) 60% and e) 90% of the maximum fluid volume. f) Measured peak‐to‐peak AP amplitudes and g) calculated SNRs in four electrode channels with different injected fluid volumes (60%, 70%, 80%, and 90% of the maximum fluid volume; *n* = 6).

**Figure** **2**a shows the impedance at 1 kHz and the CSC after the first, second, and third injections with 0%, 60%, and 90% of the maximum fluid volume, demonstrating the reusability of the developed device. After the first fluid injection, the samples used for electrochemical characterization were dried and deflated at room temperature for one day, and the fluid was reinjected. The impedance decreased after emptying the balloons and increased again after the next fluid injection. However, the CSC did not change much throughout the deflation and fluid reinjection. After the third injection with up to 90% of the maximum injectable fluid volume, the impedance and CSC tended to converge to consistent values of ≈0.9 kΩ and 0.28 mC cm^−2^, respectively. Such convergence in impedance and CSC implies that the metal patterns of the developed electrodes were intact even after repetitive fluid injection.

The impedance results were modelled according to the injected fluid volume using an adsorption equivalent circuit model. Details of the equivalent circuit modelling can be found in Figure S8 (Supporting Information). Figure 2b shows the changes in impedance at 1 kHz and CSC after soaking the electrodes in phosphate‐buffered saline (PBS) solution for 4 weeks, validating their long‐term stability. Most changes in impedance and CSC were observed during the first week and then converged. After the developed device was immersed for 4 weeks, the impedance increased by 7% and the CSC decreased by 31% compared to the initial values at week 0. Figure 2c shows the changes in impedance at 1 kHz and CSC after cyclic bending up to 1000 times by repeated fluid injection and release (Video S1, Supporting Information). After 1000 bending cycles, the impedance decreased by 18% and the CSC increased by 80% compared to the initial values. Moreover, after cyclic bending up to 1000 times, the electrodes were demonstrated to be intact, and their electrical performance was even improved. These changes in impedance and CSC are speculated to be due to an increase in the electrodes’ effective contact area with solution after fluid injection and balloon expansion.

### 2.3 Recording Performance Depending on the Injected Fluid Volume

The SACE were used to record emulated action potentials (AP) using an agarose cylinder model with a 2 mm diameter (Video S2, Supporting Information). Figure 2d,e shows the three types of sorted and averaged AP waveforms after injection with 60% and 90% of the maximum fluid volume, respectively. The noise level of the recorded APs was reduced significantly as the electrodes had secure contacts with the nerve model. Figure S9 (Supporting Information) shows the recorded APs after injection with 60% to 90% of the maximum fluid volume. The AP's peak‐to‐peak amplitude was measured, and the SNR was extracted to quantitatively analyze how recording performance changed with injected fluid volume (Figure 2f,g, respectively). Enhanced contact facilitated the recording of APs with relatively large peak‐to‐peak amplitudes and SNRs at all four electrodes. The peak‐to‐peak amplitudes averaged over all electrodes were 28.3 ± 3.4, 32.4 ± 3.4, 33.9 ± 3.7, and 36.6 ± 2.9 µV, resulting in SNRs of 6.54 ± 0.15, 7.10 ± 0.25, 7.85 ± 0.28, and 8.02 ± 0.25 after injection with 60%, 70%, 80%, and 90% of the maximum fluid volume, respectively. Consequently, larger injected volumes allowed the electrodes to contact the nerve better, improving recording performance. Therefore, injection with 90% of the maximum injectable volume was used in the in vivo experiments.

### 2.4 Recording of CNAP Responses

**Figure 3:**
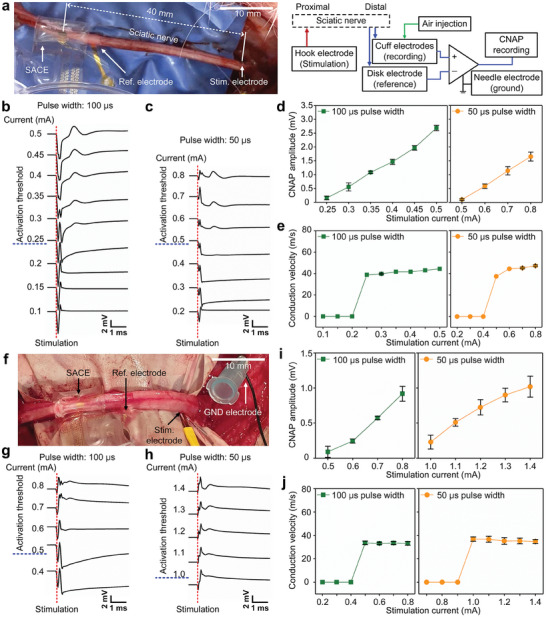
In vivo recording of CNAP responses. a) Configuration of the working, reference, and stimulation electrodes used to demonstrate the recording performance. The distance between the recording and stimulation electrodes was 40 mm. b, c) The averaged CNAP responses were measured with stimulation with pulse widths of b) 100 µs or c) 50 µs (*n* ≥ 50). Red‐dashed lines indicate the stimulation onset, and blue‐dashed lines indicate the current nerve activation threshold. To analyze the evoked CNAPs, d) the measured amplitude and e) calculated conduction velocity are presented according to stimulation with different pulse amplitudes and widths (*n* ≥ 50). Green squares and yellow circles indicate the characteristics of the CNAPs induced by stimulation using pulse widths of 100 and 50 µs, respectively. f) The same CNAP recording experiment was performed with the sciatic nerve of a canine, which has a larger nerve size. g, h) The averaged CNAP responses were measured with stimulation with pulse widths of g) 100 µs and h) 50 µs (*n* ≥ 60). i) The amplitude and j) conduction velocity of the recorded CNAPs were analyzed (*n* ≥ 60).

The feasibility of the SACE for in vivo recording was validated by recording CNAPs from a rabbit sciatic nerve (**Figure** **3**a). The CNAP responses recorded from the four electrode channels, according to the stimulation of the nerve 40 mm proximal from of the developed device with periodic biphasic pulses of 0.5 mA and 100 µs at 1 Hz (Figure S10a, Supporting Information). Likewise, Figure S10b (Supporting Information) shows the CNAP responses according to the stimulation with current pulses of 0.8 mA and 50 µs at 1 Hz. In addition, CNAP responses were examined by increasing the current amplitude from 0.1 to 0.5 mA in 0.05 mA steps with a fixed pulse duration of 100 µs (Figure 3b). Similarly, with a fixed pulse duration of 50 µs, the current amplitude was increased from 0.2 to 0.8 mA in 0.1 mA steps (Figure 3c). The amplitude of evoked CNAPs increased with stimulation intensity (i.e., injected charge) (Figure S11, Supporting Information). In addition, we confirmed that the activation thresholds were 0.25 and 0.5 mA in the stimulation with pulse widths of 100 and 50 µs, respectively; stimulation with a twofold longer pulse width required half the stimulation amplitude for nerve activation. Consequently, 25 nC was required for nerve activation.

To quantitatively analyze the evoked CNAPs, Figure 3d,e shows the measured amplitude and calculated conduction velocity, respectively, in response to various stimulation currents with different pulse widths. The CNAP response induced by stimulation with a 100 µs pulse width had amplitudes similar to those induced by twofold stronger stimulation with a 50 µs pulse width. In addition, the conduction velocity (≈40 m s^−1^) did not differ significantly with stimulation at large current amplitudes. Moreover, the evoked CNAPs were also recorded using the device located 20 mm from the stimulation site (Figure S12, Supporting Information). An evoked CNAP with an amplitude of 0.59 mV and a conduction velocity of 39.2 m s^−1^ was recorded with a stimulation pulse of 0.3 mA and 100 µs at 1 Hz. These values agreed with those recorded with stimulation using the same pulse but a 40 mm distance between the stimulation and recording electrodes. In addition, the conduction velocity of 39.2 m s^−1^ from the recorded CNAP was similar to published results.^[^ ^38^ ^]^ Furthermore, we examined the developed device's recording ability with the canine sciatic nerve, which is much larger (diameter: ≈3.5 mm) than the rabbit's (Figure 3f). Interestingly, the activation threshold of the canine nerve was 50 nC, twice the threshold for the rabbit nerve (Figure 3g,h). It showed the increasing CNAP amplitude and constant conduction velocity with larger stimulation currents as the same with the rabbit nerve (Figure 3i,j).

**Figure 4:**
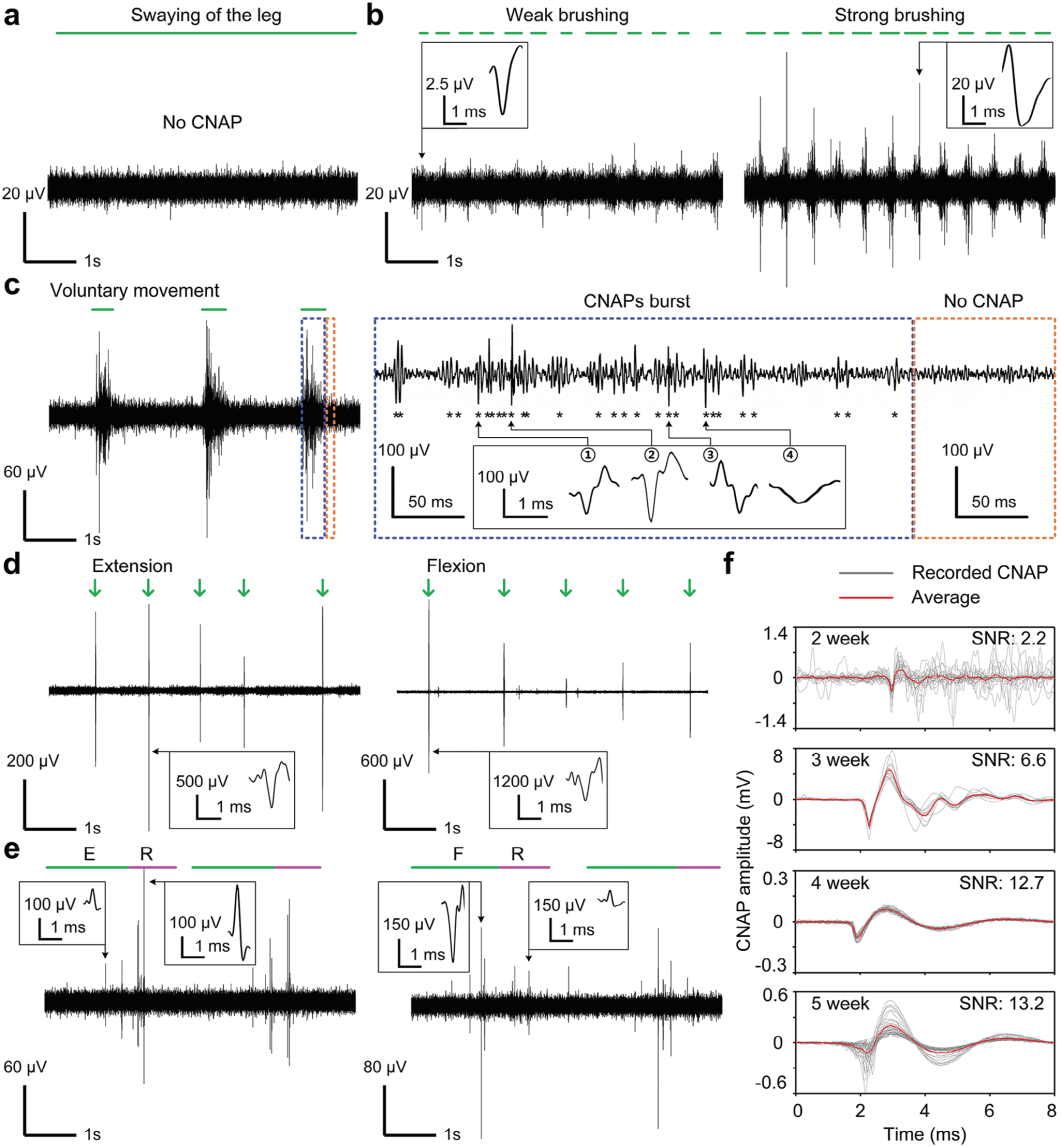
Chronic recordings of sensory and motor feedback signals from the SACE. a) As the control, no CNAPs were recorded by swaying of the leg. b) Sensory feedbacks were recorded upon tactile sensation by brushing the leg with different touch intensities. The tactile sensation and releasing periods are indicated by green and magenta lines, respectively. c**–**f) Motor feedback signals were acquired according to the duration of different leg movements. c) During the voluntary movement, a CNAP burst (blue‐dashed box) was observed, compared to the period without a CNAP (orange‐dashed box). The asterisks indicate the recorded spikes during the CNAP burst. CNAPs were recorded from d) short‐term (1 s) extension and flexion and e) long‐term (3 s) extension (denoted by E) and flexion (denoted by F) with releasing moments (denoted by R). The green arrows and lines indicate the moment and period of various leg stimulations, respectively. The magenta lines indicate the releasing period. f) The SNR of CNAP responses was measured from the results of short‐term flexion depending on device implantation time (2–5 weeks, *n* ≥ 13). The gray lines represent all 25 recorded CNAPs and the red lines represent their average.

For long‐term recording, the developed device was implanted on a rabbit's sciatic nerve for 5 weeks and recorded CNAP responses following various stimuli such as brushing, extension, and flexion. To confirm that CNAPs originated from sensory or motor fibers and not from movement artifacts, we swayed the rabbit's leg without muscle contraction, which evoked no signals (**Figure** **4**a; Video S3, Supporting Information). Sensory signals in the form of CNAPs were elicited by brushing (Figure 4b). Continuous spikes were observed during brushing, with relatively larger amplitudes detected during strong brushing. Subsequently, motor signals were recorded during various leg movements, such as voluntary movement, and forced extension and flexion. First, when anesthesia was almost over, a slight touch of the foot triggered voluntary leg movement and induced intense CNAP spikes (Figure 4c; Video S4, Supporting Information). Second, forced extension and flexion over a short distance (≈2 cm) for a short period (1 s) generated a substantial spike with large amplitudes up to 500 µV (Figure 4d; Video S5, Supporting Information). In contrast, forced extension and flexion over a longer distance (≈10 cm) for a longer period (3 s) caused consecutive spikes with relatively small amplitudes under 300 µV (Figure 4e; Video S6, Supporting Information). In addition, the spike amplitude during release was larger than during extension, whereas the spike amplitude during release was smaller than during flexion. Moreover, during flexion in a short period, the SNR was continuously increased up to 13.2 until week 5 (Figure 4f). This observed increase in SNR implies that the implanted device became more stabilized in the body, establishing better contact with the nerve over time. Furthermore, the SACE device showed not only relatively low noise level (≈16 µV) but also the highest SNR (≈13.2) compared to previous cuff electrodes (Figure S13, Supporting Information). In particular, 3D convex structures provided the SACE device with the superiority for chronic in vivo recording.

### 2.5 Neuromodulation of Peripheral Nerves

**Figure 5:**
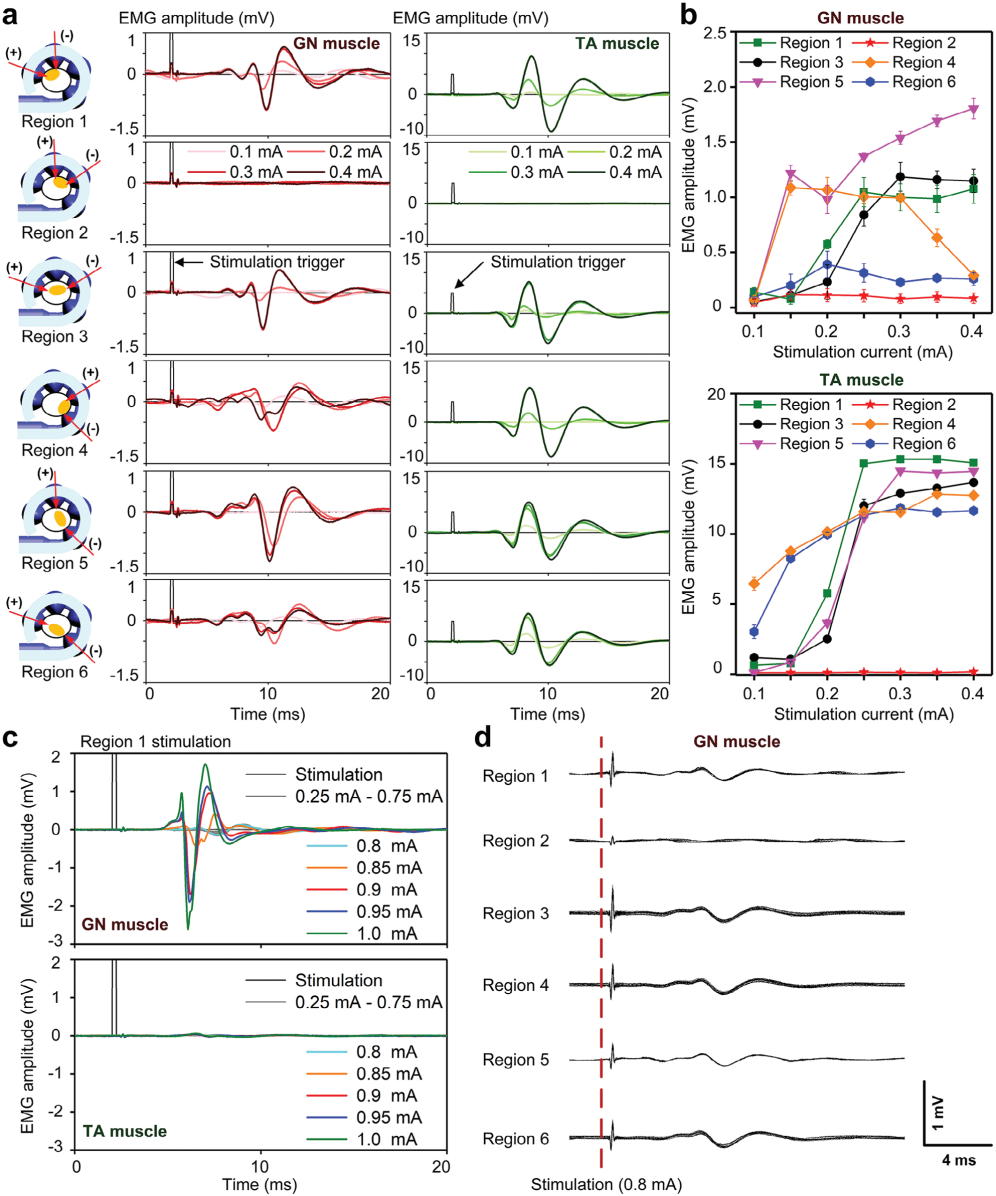
EMG responses induced by acute and chronic nerve stimulation depending on the stimulation regions. a) Six regions of the nerve were stimulated by six different combinations of the four channels of the SACE. The black lines indicate the trigger signal for stimulation. EMG responses were recorded from the GN and TA muscles upon nerve stimulation with amplitudes ranging from 0.1 to 0.4 mA (*n* = 20). b) To analyze the EMG responses detected from GN and TA muscles, the amplitude of evoked EMGs is presented for different stimulation currents and regions (*n* = 20). To validate the long‐term stimulation, EMG responses from GN and TA muscle were measured after c) 2 weeks and d) 6 weeks of device implantation (*n* = 25). In c), only the GN muscle was activated by only region 1 stimulation, indicating selective stimulation. On the other hand, in (d), EMG responses were evoked upon stimulation in all regions except region 2, indicating improved stimulation performance at week 6 than at week 2.

The developed SACE device was used to stimulate the sciatic nerve while recording EMG responses from the GN and TA muscles (Figure S14, Supporting Information). The sciatic nerve was stimulated with pulse amplitudes of 0.1, 0.2, 0.3, and 0.4 mA through six different regions determined by the six combinations of cathode and anode pairs among the four electrode channels of the developed device (Figure S15, Supporting Information). **Figure** **5**a shows the averaged EMG responses from the GN and TA muscles induced by nerve stimulation. The EMG responses were observed when stimulating all regions except region 2, demonstrating the possibility of selective stimulation. Figure 5b indicates that the amplitude of evoked EMG responses from the GN and TA muscles depends on the stimulation current. For the GN muscle, various tendencies of the change in EMG amplitude were observed as the stimulation current increased. The decrease in EMG amplitude with regions 4 and 6 was due to the EMG peak splitting into two, reducing its amplitude. In contrast, the EMG amplitude was saturated with all regions for the TA muscle.

When large stimulation amplitudes up to 1 mA were applied, the evoked EMG response tended to increase in both the GN and TA muscles (Figures S16a and S17, Supporting Information). The GN and TA muscles were most activated when a stimulation amplitude of 1 mA was exerted to each of the six regions. Interestingly, the GN muscle was highly activated, resulting in larger EMG responses with amplitudes >25 mV upon nerve stimulation in regions 5 and 6. The EMG response was sevenfold larger from the GN muscle than the TA muscle when both muscles were highly activated upon stimulation with a 1 mA current. This difference might be because the GN muscle contains more muscle fibers (>10 00 000 filaments) than the TA muscle (2 70 000 filaments).^[^ ^39^ ^]^ In addition, we could identify the location of a common peroneal nerve governing the TA muscle and a tibial nerve governing the GN muscle, through the analysis of EMG responses without incision of the nerve (Figure S16b, Supporting Information).

Long‐term stimulation was validated by observing EMG responses from GN and TA muscles after 2 and 6 weeks of implantation (Figure 5c,d, respectively). After 2 weeks of implantation, the nerve was stimulated using currents from 0.25 to 1 mA with an interval of 0.05 mA. Only the GN muscle was activated and the EMG responses started to be recorded by stimulation with a large current ≥0.8 mA (Figure 5c). The amplitude of the evoked EMG response increased with the stimulation current, but only stimulation at region 1 could activate the GN muscle after 2 weeks of device implantation. On the other hand, stimulation performance was enhanced after 6 weeks of device implantation. EMG responses and leg twitching were observed upon stimulation using smaller current pulses of 0.4–0.8 mA (Video S7, Supporting Information). In addition, EMG responses were evoked by the stimulation of all regions except for region 2. (Figure 5d). We speculated that the performance of chronic nerve stimulation was enhanced following the stabilization of the device together with the surroundings inside the body. Furthermore, we could achieve selective stimulation by observing different responses. When the GN muscle was activated, the backside of the leg twitched and the leg was extended (Video S8, Supporting Information). In contrast, when the TA muscle was activated, leg flexion was caused (Video S9, Supporting Information). When both GN and TA muscles were activated simultaneously, strong twitching of the whole leg, rather than leg extension or flexion, was caused (Video S10, Supporting Information).

### 2.6 Validation of Minimal Nerve Damage

**Figure 6:**
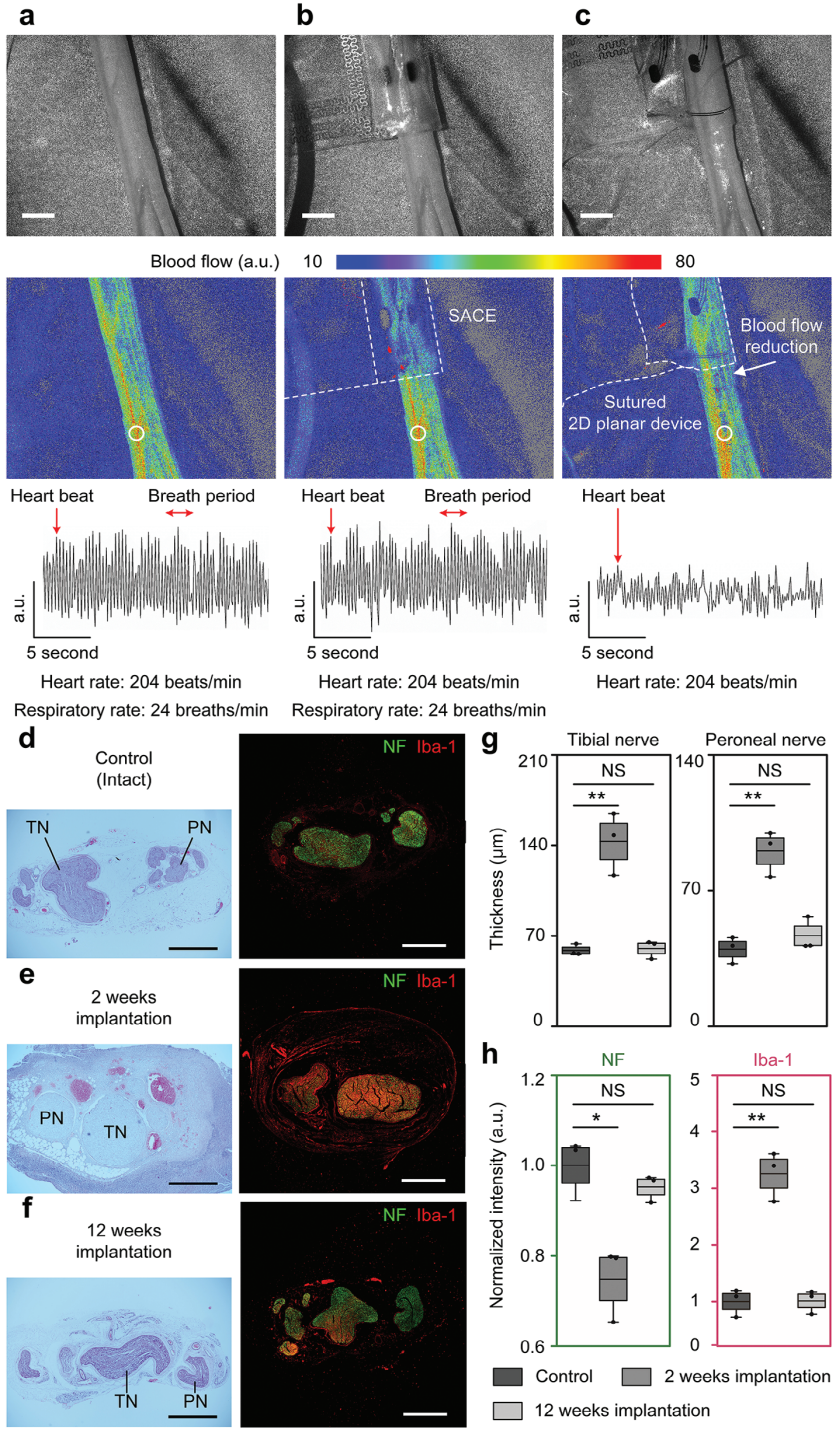
Demonstration of minimal nerve damage through blood flow measurement and nerve histology after device implantation. The blood flow on the nerve surface was measured by 2D laser speckle imaging and 1D laser Doppler flowmetry a) without any device implantation, b) after implantation of the SACE by fluid injection, and c) after suturing conventional 2D planar device. The black‐and‐white images show the nerve with or without device implantation. The 2D speckle images show the blood flow for the nerve with or without implanted devices shown as white‐dashed lines. A reduction in blood flow was observed next to the boundary of the sutured device, whereas no reduction in blood flow was observed for the fluid‐injected device. From the relative blood flow indices, the blood flow with the heartbeat and breath period was measured at the white circles in the 2D speckle images. A heart rate of 204 beats min^−1^ and a respiratory rate of 24 breaths min^−1^ were observed from the blood vessel on the nerve surface implanted with no device and with SACE. However, the respiratory rate could not be detected from the nerve implanted with sutured 2D planar device due to the lowered blood flow. d–f) The nerve histology was performed by H&E and IHC staining. Three groups of nerve samples were compared: d) the control, e) the sample after 2 weeks of device implantation, and f) the sample after 12 weeks of device implantation. In H&E staining, cell nuclei are stained blue‐purple, and the extracellular matrix and cytoplasm are stained pink. The areas stained in red correspond to the blood vessels. In IHC staining, green and red fluorescence indicates neurofilaments (NF) and macrophages (Iba‐1) in nerve samples, respectively. The scale bars are 2 mm and 500 µm in the blood flow and nerve histology results, respectively. For quantitative analysis, g) the thicknesses (*n* = 3) of connective tissues around tibial nerve (TN) and peroneal nerve (PN) and h) the normalized fluorescent intensities (*n* = 3) of NF and Iba‐1 were measured. Statistical significance and *p*‐values are calculated by two‐sided unpaired *t*‐test (NS: not significant, ^*^: *p* < 0.05, ^**^: *p* < 0.01).

The contact pressure between the electrodes and the sciatic nerve was evaluated by analyzing blood flow (Figure S18a, Supporting Information). The blood flow after SACE implantation based on fluid injection was compared to that after suturing conventional 2D cuff device (Figure S18b,c, Supporting Information). **Figure** **6** shows the images of a nerve with or without the implanted device, 2D laser speckle images of blood flow, and 1D Doppler flowmetry of relative blood flow index. As a control group, blood flow was measured for nerves with no implanted device (Figure 6a). From the relative blood flow index, the heart rate and respiration rate could be derived from the peaks in the blood flow and the sinusoidal waveform caused by breathing, respectively. A heart rate of 204 beats per min was detected, which is common for a normal rabbit under anesthesia.^[^ ^40^ ^]^ A respiration rate of 24 breaths min^−1^ was observed, the same as the controlled ventilation rate for anesthesia. Figure 6b shows the blood flow after implantation of the SACE. Compared with the blood flow in the control group, there was no difference in blood flow from the speckle image or in the heart and respiratory rates based on the measured relative blood flow index at a specific location denoted by a white circle. On the other hand, the blood flow of the nerve sutured with 2D planar device was 55% lower than the control group (Figure 6c). It was apparent that suturing the device mechanically pressed the nerve and affected blood flow. While the heart rate was measured to be 204 beats per min, the same as in the control group, the respiratory rate was undetectable because the blood flow intensity was too low.

Hematoxylin and eosin (H&E) staining and immunohistochemistry (IHC) staining of the implanted nerves were performed to investigate any possible nerve damage after long‐term device implantation. Three groups of control, device after 2 weeks of implantation, and device after 12 weeks of implantation were prepared and compared in the aspects of morphology and cell distribution. The control samples were acquired from intact sciatic nerves where surgery was performed to expose the nerve, but no device was implanted (Figure 6d). The other samples were acquired from sciatic nerves where the device had been implanted for 2 weeks (Figure 6e) and 12 weeks (Figure 6f). H&E staining showed that nerves with the device implanted for 12 weeks had similar epineurium and perineurium morphologies to the control nerves. Notably, epineurium and perineurium shrinkage was observed during the paraffin exchange because of little connective tissues created by inflammation reaction and swelling. In contrast, for nerves with the device implanted for 2 weeks, most of the area between the epineurium and perineurium was filled with connective tissues, which also wrapped the epineurium in a thick layer. Due to these connective tissues, the perineurium of fascicles showed circular shapes, even after the paraffin exchange process. In addition, large‐sized blood vessels were distributed inside the nerve, indicating inflammation reactions. The thicknesses of connective tissues formed around tibial nerve and peroneal nerve were quantified (Figure 6g). For both nerves, the thickness of connective tissues was similar in the control and 12 weeks groups. On the other hand, it was more than twice in the 2 weeks group. IHC staining showed the cell distributions: neurofilament (NF), macrophage (Iba‐1), myelin (maltose binding protein, MBP), and nuclei (DAPI) (Figure S19, Supporting Information). The control nerve (Figure 6d) and the nerve implanted with the device for 12 weeks (Figure 6f) similarly showed abundant neurofilaments and less macrophages, indicating that the distribution of nerve cells after 12 weeks of implantation was comparable to that with no implanted device. In contrast, the nerve implanted with the device for 2 weeks showed less neurofilaments and abundant macrophages (Figure 6e). Fluorescent intensities of NF and Iba‐1 were normalized and compared among groups (Figure 6h). NF and Iba‐1 intensities were similar in the control and 12 weeks groups. However, in the 2 weeks group, NF intensity was lower by 25% whereas Iba‐1 intensity was more than three times higher than the control. Notably, macrophages were abundant around the entire area of the nerve, including inside the nerve fascicles and outside the epineurium. This temporal change in cell distribution would implicate that biological responses in the nerve are progressive during weeks immediately after device implantation. However, the nerve recovers after a sufficient period of time, suggesting the promising potential for longer‐term use of the SACE device.

## 3 Discussion and Conclusion

We developed the SACE that can envelop the nerve using a pneumatic‐actuated method. Though several actuation mechanisms for enveloping nerves by using voltage,^[^ ^24^ ^]^ water,^[^ ^32^ ^]^ or heat^[^ ^16^ ^]^ treatment have been successfully developed, secured contact to nerves has remained an open question. On the other hand, our new paradigm to implant cuff devices on nerves enables minimal and secure contact between the electrodes and the nerve thanks to their unique 3D convex structures. The injected fluid volume could precisely control the bending diameter, and the developed device could envelop the nerve with a low contact force of <1.21 gf, which is indeed the first to report the contact force between a cuff device and the nerve. The excellent mechanical properties of the SACE device have been demonstrated by measuring the contact pressure of the device to nerves. The contact force of the device to nerves with a diamter of 2.5 mm was measured to be 0.9 gf (Figure S6, Supporting Information) and the minimal contact area to nerves can be supposed as similar to the electrode area (≈2.33 mm^2^) of the SACE device. Then, the pressure on nerves is calculated to be 3.78 kPa, which is acceptable for an implanted device because 4.0 kPa of pressure is known to start degrading nerve functions.^[^ ^41^ ^]^ On the other hand, 1.3 kPa of pressure on nerves is known to show little axonal degeneration or demyelination even after chronic nerve compression for several weeks. Therefore, when we suppose the contact area of the SACE device as half the balloon area (≈8 mm^2^), the pressure on nerves would be only 1.1 kPa, by which minimal nerve damage can be assured. Furthermore, Channel 1 in Figure S11 (Supporting Information) was the channel farthest away from the injection site. The signal amplitude detected by Channel 1 was similar to those detected by other channels, indicating that the pressure by the protrusion farthest from the injection site was also sufficient to form secure contact with the nerve.

Moreover, the developed device might be applied to diverse sized human nerves by adjusting the device parameters, especially the dimensions and number of balloons (Figure S20, Supporting Information). In addition, the electrochemical characterization showed that the developed device consistently maintained the impedance and CSC over repeated fluid injections, demonstrating its reusability, stability, and repeatability. Furthermore, injecting 90% of the maximum fluid volume produced firm contact between the electrodes and the nerve, resulting in better recording performance with increased AP amplitude (by 29.3%) and SNR (by 22.6%) compared to injection with 60% of the maximum fluid volume. Secure contact of all electrodes was guaranteed when the bent device had a smaller ID than the nerve diameter.

To demonstrate the use of the developed SACE for stimulation and recording of peripheral nerves, in vivo experiments were conducted on the sciatic nerves of rabbits or canines. While the nerve diameter and shape vary depending on the subject, the developed device demonstrated secure contact with the nerve, confirmed by electrical connection of all four electrode channels. In both sciatic nerve stimulation and recording experiments, a larger stimulation amplitude caused a large and fast response of the evoked CNAP and EMG. The nerve conduction velocity is known to depend on the axon diameter inside the nerve.^[^ ^42^ ^]^ Axons with larger diameters require a smaller stimulation current for activation and enable faster conduction of APs. Consequently, the large amplitude and fast conduction velocity could be because many axons with relatively large diameters have been activated by increasing the stimulation current.^[^ ^43^, ^44^, ^45^ ^]^ In addition, while a biphasic pulse was used for stimulation to cancel out the stimulation artifacts in the recorded CNAP response,^[^ ^42^ ^]^ it was harder to eliminate the artifacts when a longer pulse width of 100 µs was used compared to a shorter pulse width of 50 µs (Figure 3b,c). The degree of activation for GN and TA muscles differed depending on the spatially selective stimulation of the nerve in the circumferential direction. Accordingly, the locations of tibial and common peroneal nerves innervated by the sciatic nerve could be estimated through the EMG responses from GN and TA muscles to selective nerve stimulations. Furthermore, the developed SACE successfully demonstrated long‐term stimulation and recording for 6 weeks. Both sensory and motor signals from the sciatic nerve could be acquired upon mechanical stimuli on the skin and forced leg movement, respectively. While long‐term experiments demonstrated selective stimulation, EMG responses were induced only in the GN muscle. We speculated that this finding could reflect the animal's unhealthy condition during the experimental period. Because rabbits are vulnerable to changes in their external environment, they starved themselves and it was hard to find the exact location of the TA muscle.

By examining the blood flow in the nerve, we demonstrated that the developed SACE device preserved the nerve intact, which showed no reduction in blood flow, unlike the 2D planar cuff device implanted by conventional suturing. In addition, the histological analysis at 12 weeks after device implantation showed minimal nerve damage by the SACE (Figure 6f). The nerves wrapped by the developed device appeared to recover over time during implantation. Furthermore, the SACE clearly demonstrated (Figure S21, Supporting Information) that there were no significant foreign body responses and subsequent fibrosis around the device after 7‐months implantation. The interface between the electrodes and nerve surface was clearly differentiable without connective tissues formed around, thereby indicating the biological stability of the SACE.

In conclusion, the SACE device is expected to be appropriate for implantation with minimally affecting the nerve while securing contact between the electrodes and the nerve. The soft 3D convex structures reduced the contact area between the electrodes and the nerve with minimal contact force, thereby causing minimal foreign body responses, such as inflammation and fibrosis. Since secure contact between the electrodes and the nerve could be achieved with only a small amount of force generated by fluid injection‐based soft actuation, this simple and convenient implantation mechanism would allow the developed electrodes to minimally affect the nerve. Long‐term experiments successfully demonstrated the recording of sensory and motor CNAP signals with superior SNRs up to 13.2 as well as selective nerve stimulation over weeks. This high SNRs clearly demonstrated the advantage of the proposed 3D structure, compared to conventional cuff electrodes embedded in 2D sheets whose SNRs range from 3 to 10.^[^ ^16^, ^34^, ^46^, ^47^, ^48^, ^49^, ^50^ ^]^ (Figure S13, Supporting Information). In addition, minimal nerve damage during long‐term implantation was confirmed as the suggested implantation method did not reduce blood flow in the nerve nor alter the cell distribution in the nerve after 12 weeks of implantation. Thanks to the soft and flexible materials used and simple implantation method based on soft actuation, we expect the developed device to be a promising alternative in bidirectional neuroprostheses for stimulating and recording peripheral nerves.

## 4 Experimental Section

### Fabrication of the SACE

The electrodes device comprised three layers of a 200 µm‐thick bottom PDMS layer (Sylgard 184, Dow Corning Corp.), a 500 nm‐thick middle parylene C layer (Parylene C dimer, Nuri‐Tech Corp.), and a 100 µm‐thick top PDMS layer. For selective bonding between the parylene C and top PDMS layer, a 250 nm Ti masking layer was deposited by a RF‐biased sputter (SRN‐130, SORONA Inc.) and patterned using photolithography. The substrate surface was treated with plasma of nitrogen and oxygen mixed gas using reactive ion etching (RIE; VITA, Femto Science). The RIE conditions used were a nitrogen flow rate of 200 sccm, an oxygen flow rate of 10 sccm, a power of 150 W, and a treatment time of 30 min. After removing the Ti mask, 1 µm‐thick parylene C was deposited to mitigate the mechanical mismatch between the PDMS substrate and metal electrodes.^[^ ^14^, ^51^ ^]^ Then, Ti/Au layers of 25/300 nm thicknesses were deposited using a DC sputter (SRN‐110, SORONA Inc.) and patterned to form the electrodes, conductive lines, and connection pads. To insulate the conductive lines, 4.3 µm‐thick parylene C was deposited by low‐pressure chemical vapor deposition (NRPC‐500, Nuri‐Tech Corp.) and patterned by RIE with oxygen plasma (oxygen flow rate of 20 sccm, 50 W, and 160 min). For long‐term nerve stimulation, iridium oxide was electrodeposited onto the Au electrodes to enhance the CSC. The Au electrodes were immersed in the solution (0.15 w/w% iridium chloride, 0.5 w/w% oxalic acid, and 8 w/w% potassium carbonate mixed with DI water) and a cyclic voltage ranging from 0 to 0.8 V was applied 50 times at a scan rate of 25 mV s^−1^. Two PDMS blocks with dimensions of 5 × 5 mm^2^ were bonded onto both sides of the top and bottom PDMS layers, which had a punched hole, and a silicone tube was inserted into the upper PDMS block. Air with a volume of 459.7 µL was injected into the fluidic channel at a flow rate of 170 µL min^−1^ using a syringe pump (Fusion 100, Chemyx Inc.) to create a bent shape of the developed device (see fabrication details in Figure S1, Supporting Information). For long‐term recording and stimulation, PDMS was injected into the device and cured in an oven at 55 °C for 2 h for shaping a permanent 3D structure.^[^ ^14^ ^]^

### Measurement of Injected Fluid Volume

Figure S2a (Supporting Information) shows the force measurement setup during fluid injection. A force meter (ZTA‐50N, IMADA Co.) was mounted onto an XY‐stage (Cross 130‐HSM, OWIS GmbH), and a syringe was held in a customized holder in front of the force meter. A silicone tube connected the syringe to the fluidic channel of the developed device (Figure S2b, Supporting Information). For fluid injection with force measurement, the force meter pushed the syringe's piston at 200 µm s^−1^. When the fluidic channel burst, the amount of injected fluid was considered the maximum injectable fluid volume. Ninety percent of the maximum injectable fluid volume was used subsequently to stably bend the device (Figure S2c, Supporting Information).

### Electrochemical Characterization

Electrochemical impedance spectroscopy (EIS) and CV of the developed device were performed before and after fluid injection, using a potentiostat/galvanostat (Reference 600+, Gamry Instruments). A conventional three‐electrode setup was used: the developed electrodes were the working electrode, a Pt electrode was the counter electrode, and an Ag/AgCl electrode was the reference electrode. These three electrodes were immersed in PBS (pH 7.4) solution in a Faraday cage. The EIS was performed from 10 Hz to 100 kHz with an AC excitation of a 30 mV_rms_ sinusoidal signal. The CV measurement used a scan rate of 100 mV s^−1^ with potential limits of −0.6 and 0.8 V. The CSC was calculated from the CV curves using the following equation:

(1) $$\mathrm{CSC}\;\left(mC/cm^{2}\right)=\frac{1}{\nu }\;\int_{Ec}^{Ea}\left|J\right|dE$$

where ν, *E*_a_, *E*_c_, *J*, and *E* are the scan rate (V/s), the potential limit of the anode (V), the potential limit of the cathode (V), the measured current density (A/cm^2^), and the potential versus the reference Ag/AgCl electrode (V).

### Validation of Electrical Contact Using the Agarose Nerve Model

The electrical contact between the electrodes and the nerve was examined by analyzing recording performance depending on the injected fluid volume. The experimental setup included an agarose nerve model, the developed device, and an AP emulator (Digital Neural Signal Simulator, BlackRock Microsystems).^[^ ^37^ ^]^ For the fabrication of agarose nerve model, 5 w/v% agar (Agar powder, Duksan reagents) was mixed in 5x Tris‐acetate‐EDTA buffer (50x TAE Buffer, Thermo Fisher Scientific; diluted to 1:10 in DI water) and completely dissolved using an autoclave (121 °C, 15 min). Then, the dissolved agar solution was poured in a 3D‐printed mold for nerve shaping and cooled in the ambient condition for 10 min. Each side of the agarose nerve model was electrically connected to the signal output and ground of the emulator through conducting wires. Three AP waveforms were repeatedly injected into the agarose nerve model with a diameter of 2 mm. The emulated AP signals were recorded through the SACE electrodes for different fluid injection volumes (60%, 70%, 80%, and 90% of the maximum injectable volume). The SNR of the recorded AP was calculated using the following equation:

(2) $$\mathrm{SNR}\;=\frac{V_{\mathrm{pp}}}{2*\sigma_{\mathrm{noise}}}\;$$

where *V*_*pp*_ and σ_*noise*_ are the peak‐to‐peak amplitude of AP and the standard deviation of noise, respectively. SNRs were calculated from emulated APs without using filter (Figure 2g) and from evoked CNAPs after applying 150–5000 Hz band‐pass filter (Figure 4f), respectively.

### Surgery to Expose the Sciatic Nerve

Surgical procedures for rabbits and canines were conducted in compliance with the guidelines approved by the Institutional Animal Care and Use Committee of Daegu Gyeongbuk Institute of Science and Technology (approval no. DGIST‐IACUC‐22120201‐0004) and Osong Medical Innovation Foundation (approval no. KBIO‐IACUC‐2022‐052‐1), respectively. Six adult New Zealand white rabbits (male, 12 weeks old, weighing ≈5 kg) and one adult canine (male, three years old, weighing ≈13.4 kg) were used. All surgical instruments, including the SACE, parylene C‐based disk reference electrodes, needle ground electrodes, hook stimulation electrodes, and needle‐type EMG electrodes, were sterilized using a 70% ethanol solution before the surgery. An endotracheal tube (ID: 3 mm) was inserted into the bronchial tube of the rabbit or canine for anesthesia. The anesthetic gas of 5% isoflurane (Isotroy 100, Troikaa Pharmaceuticals Ltd.) in oxygen was delivered at a flow rate of 2 L min^−1^ for initial anesthesia. After the animal's breathing became comfortable, the anesthesia was maintained with 2% isoflurane in oxygen at a flow rate of 1 L min^−1^. The site for surgery was shaved and sterilized using 70% ethanol and iodine solutions. Then, an incision of skin and muscle around the biceps femoris was made. An incision of the fascia exposed the area between the muscles. When the sciatic nerve was visible, we opened the surrounding muscles widely and removed the surrounding tissues around the nerve. A piece of latex sheet was placed under the sciatic nerve to electrically insulate it from the adjacent muscles.

### In Vivo Experiment for CNAP Recording

Upon injection with an air volume of 459.7 µL, the cuff electrodes enveloped the nerve as the working electrode. The parylene C‐based disk electrode wrapped the nerve next to the developed device as the reference electrode. A syringe needle electrode was inserted into a rabbit tail or a subcutaneous muscle unrelated to the recording site at the sciatic nerve. A tungsten hook electrode was placed 40 mm proximally from the SACE device (Figure 3a). The SACE, parylene C‐based reference electrode, and ground electrode were connected to a signal acquisition system (CerePlex direct, BlackRock Microsystems) for recording the CNAP. A stimulator (Model 2100, A‐M Systems) was connected to the hook electrode to provide stimulation. Stimulation used varying pulse amplitudes with pulse widths of 100 and 50 µs. The evoked CNAPs recorded through the developed SACE were band‐pass filtered using a fourth‐order Butterworth filter with cutoff frequencies from 150 to 5000 Hz. The amplitude was measured from a medium point between the start and end points of the evoked CNAP to a vertex. The latency was measured from the end point of the stimulation trigger to a vertex of the evoked CNAP. For chronic recording, the PDMS‐injected device was used and its 3D structures contacted the nerve surface. Then, the device grasping the nerve was fixed using a biocompatible silicone adhesive (Sil‐Poxy, Smooth‐On Inc.).

### In Vivo Experiment for Sciatic Nerve Stimulation

The implantation method of the developed device was the same as used in recoding. Among the four channels, one channel was used as the anode and another as the cathode for stimulation. Six different pairs of electrode channels were used to stimulate the sciatic nerve with a stimulator (Model 2100, A‐M Systems). Two‐needle EMG electrodes were inserted into both the GN and TA muscles. EMGs were recorded through a data acquisition system (MP36U‐W, Biopac Systems Inc.) according to nerve stimulations in various regions and of various amplitudes.

### Blood Flow Detection

The blood flow was detected by measuring the corpuscle velocity inside the blood vessel using a 1D laser Doppler flowmeter (OMEGAFLO‐Lab, OMEGAWAVE Inc.) and 2D laser speckle flow imager (OMEGAZONE OZ‐2 mini, OMEGAWAVE Inc.). The 2D laser speckle flow imager could acquire both the black and white nerve image and the blood flow speckle image. The relative blood flow index was measured by the 1D laser Doppler flowmeter for 20 s at the specific location of the blood vessel on the nerve surface indicated by the white circle (Figure 6a–c). Figure S18a (Supporting Information) shows the 2D laser speckle imaging where a CCD camera with a laser source/detector was set up, and the 1D laser Doppler flowmetry included an optical fiber fastened 2 mm from the blood vessel on the nerve surface. The CCD camera took a speckle image of blood flow for 1 s, and the optical fiber included both the laser source and detector. The laser speckle intensity scattered by the blood corpuscles flowing inside the blood vessel was visualized as a colored speckle image.

### H&E Staining

The sciatic nerve sample was immersed overnight in 4% paraformaldehyde (PFA) at 4 °C and then overnight in PBS (pH 7.4) solution at 4 °C. For paraffin sectioning, the moisture contained in the sample was exchanged for paraffin using a tissue processor (HistoCore PEARL, Leica). Then, the sample was trapped in a paraffin block using a paraffin embedding machine (HistoCore Arcadia H, Leica). The paraffin block containing the sample was sliced at 4 µm thickness using a rotary microtome (RM2255, Leica). The sliced sample was placed in a bath filled with 36 °C deionized (DI) water, then scooped and placed onto a glass slide. After drying on a 40 °C hotplate for 10 min, the glass slide containing the sample was sequentially immersed in 100%, 100%, 95%, 80%, and 70% xylene solutions and DI water for 3 min each to remove the paraffin components of the sliced sample. Then, H&E staining of the sample was performed using an H&E Staining Kit (ab245880, Abcam). The thicknesses of connective tissues around the tibial nerve and peroneal nerve were measured by using ImageJ software (National Institutes of Health).

### Immunohistochemistry

The sciatic nerve was harvested by transection. The sample was fixed overnight in a 4% PFA solution and then immersed in a solution of 30% sucrose in PBS for 36 h. Then, the sample was embedded in the optimal cutting temperature (OCT) compound (Scigen, Paramount, CA, USA) and frozen at −80 °C overnight for cryosectioning. The OCT compound block containing the sample was sliced using a cryo‐microtome (CM3050S, Leica) with a 14 µm thickness and placed onto a glass slide. For IHC staining, the glass slide containing the sample was washed four times for 5 min with PBS. Next, it was incubated with a blocking solution of PBS containing 0.3% Triton X‐100 and 5% normal donkey serum (017‐000‐121, Jackson ImmunoResearch) for 1 h at room temperature. Then, the sample was incubated overnight at 4 °C in a 5% blocking solution containing the primary antibodies mouse anti‐NF200 (N5389, Sigma‐Aldrich; 1:200 dilution), chicken anti‐MBP (PA1‐10008, Invitrogen; 1:500 dilution), or goat anti‐Iba‐1 (ab5076, Abcam; 1:50 dilution). Next, the sample was rinsed four times for 5 min with PBS. Then, the sample was stained overnight at 4 °C in a PBS solution containing 0.3% Triton X‐100 and the secondary antibodies Alexa Fluor 488‐conjugated donkey anti‐chicken (A78948, Abcam; 1:1000 dilution), Alexa Fluor cy3‐conjugated donkey anti‐goat (ab6949, Abcam; 1:1000 dilution), or Alexa Fluor 647‐conjugated donkey anti‐mouse (A11126, Abcam; 1:200 dilution). At the same time, the sample was nuclear counterstained using DAPI (ab228549, Abcam; 1:1000 dilution). Then, the sample was washed four times for 5 min with PBS and mounted using a mounting solution (P36987, Invitrogen) and a cover glass. After curing the sample at room temperature for 6 h, a fluorescent image was acquired using a confocal microscope system (C2+, Nikon). The fluorescent intensities of neurofilaments and macrophages were quantified by using ImageJ software.

### Statistical Analysis

Data obtained from this study were presented as the mean ± standard deviation unless otherwise stated. The sample size (*n*) used in each experiment was described in the figure legends. All recorded CNAP signals and EMG signals were processed using 150–5000 Hz band‐pass filter (Figures 3, 4, 5). Two‐sided unpaired *t*‐test was performed to determine the statistical significance between groups (Figure 6g,h). The difference between groups was considered statistically significant if *p* < 0.05 (NS: not significant; ^*^: *p* < 0.05; ^**^: *p* < 0.01).

## Conflict of Interest

The authors declare no conflict of interest.

## Supporting information



Supporting Information

Supplemental Video 1

Supplemental Video 2

Supplemental Video 3f

Supplemental Video 4

Supplemental Video 5

Supplemental Video 6

Supplemental Video 7

Supplemental Video 8

Supplemental Video 9

Supplemental Video 10

## Data Availability

The data that support the findings of this study are available from the corresponding author upon reasonable request.
