# Supplementary material for: Soft‐Actuated Cuff Electrodes with Minimal Contact for Bidirectional Peripheral Interfaces
Source: Adv Mater. 2024 Nov 10;37(5):2409942. doi: 10.1002/adma.202409942 (PMC11795727; doi:10.1002/adma.202409942)
Supplement: Supplementary file 1 — Supporting Information [file ADMA-37-2409942-s008.pdf]

ADVANCED MATERIALS

Supporting Information

for *Adv. Mater.*, DOI 10.1002/adma.202409942

Soft-Actuated Cuff Electrodes with Minimal Contact for Bidirectional Peripheral Interfaces

*Hyunmin Moon, Byungwook Park, Namsun Chou, Ki-Su Park, Sanghoon Lee and Sohee Kim**

Supporting Information

Soft-actuated cuff electrodes with minimal contact for bidirectional peripheral interfaces

*Hyunmin Moon, Byungwook Park, Namsun Chou, Ki–Su Park, Sanghoon Lee, and Sohee Kim**

H. Moon, B. Park, S. Lee, S. Kim
Department of Robotics and Mechatronics Engineering
Daegu Gyeongbuk Institute of Science and Technology (DGIST)
Daegu 42988, Republic of Korea
E-mail: soheekim@dgist.ac.kr

H. Moon
Department of Mechanical Engineering
Massachusetts Institute of Technology
Cambridge, MA 02139, USA

N. Chou
Emotion, Cognition, & Behavior Research Group
Korea Brain Research Institute
Daegu 41062, Republic of Korea

K.-S. Park
Department of Neurosurgery
Kyungpook National University School of Medicine
Daegu, 41944, Republic of Korea

Supporting Figures

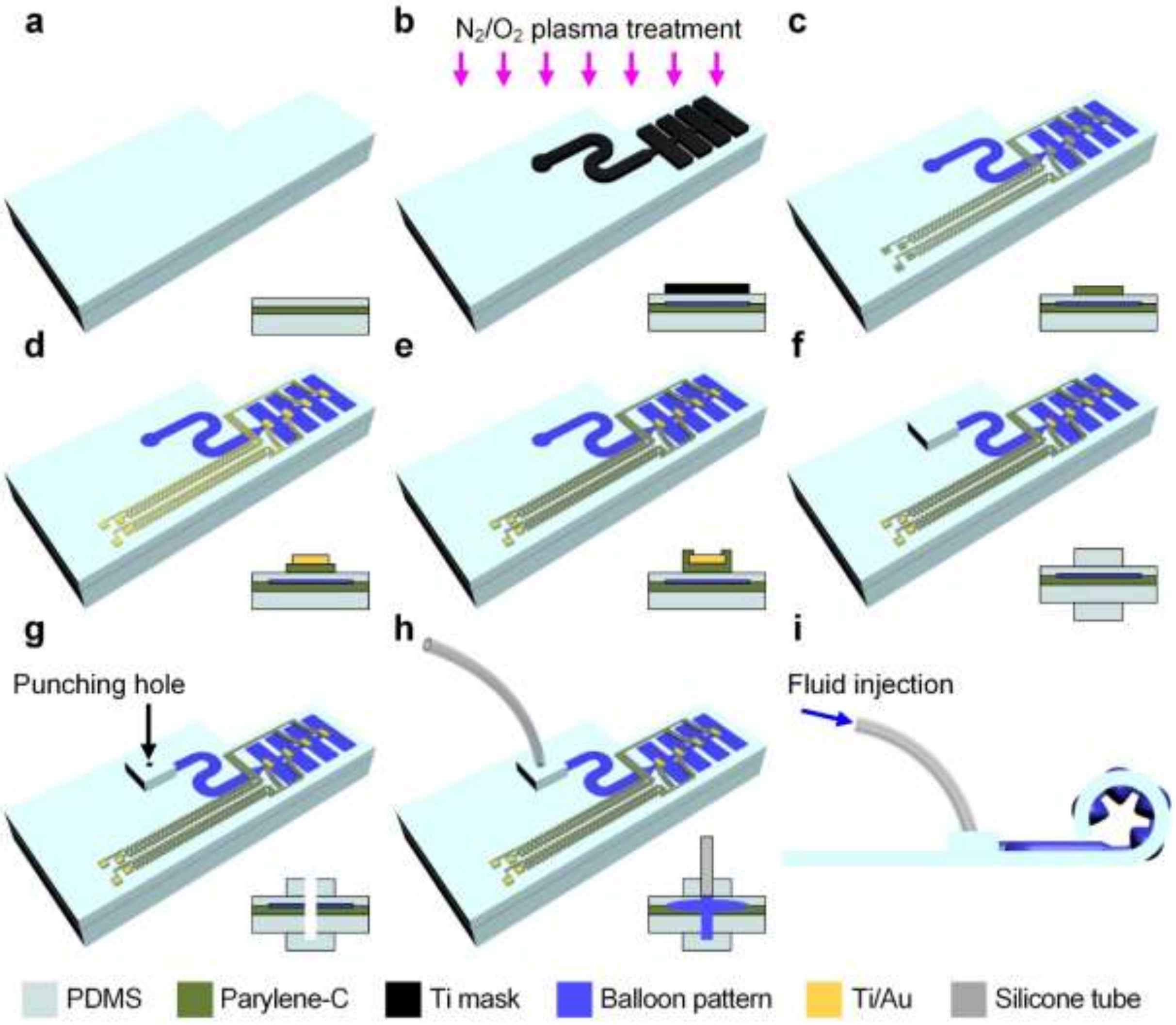

Figure S1. Fabrication of the SACE. (**a**) A substrate consisting of bottom PDMS, parylene C, and top PDMS layers was prepared. (**b**) After patterning a Ti mask on the substrate, a nitrogen and oxygen plasma treatment was applied to generate a fluidic channel. After removing the Ti mask, (**c**) parylene C and (**d**) Ti/Au layer was deposited and patterned on the substrate surface. (**e**) The insulation with parylene C layer was deposited and patterned. (**f**) Two PDMS blocks were attached onto both sides of the top and bottom PDMS layers at the inlet of the fluidic channel using an oxygen plasma treatment. (**g**) A hole was punched at the center of the top PDMS block using a biopsy punch. (**h**) A silicone tube was inserted into the top PDMS block. (**i**) A fluid was injected into the fluidic channel to generate a bent shape with balloons.

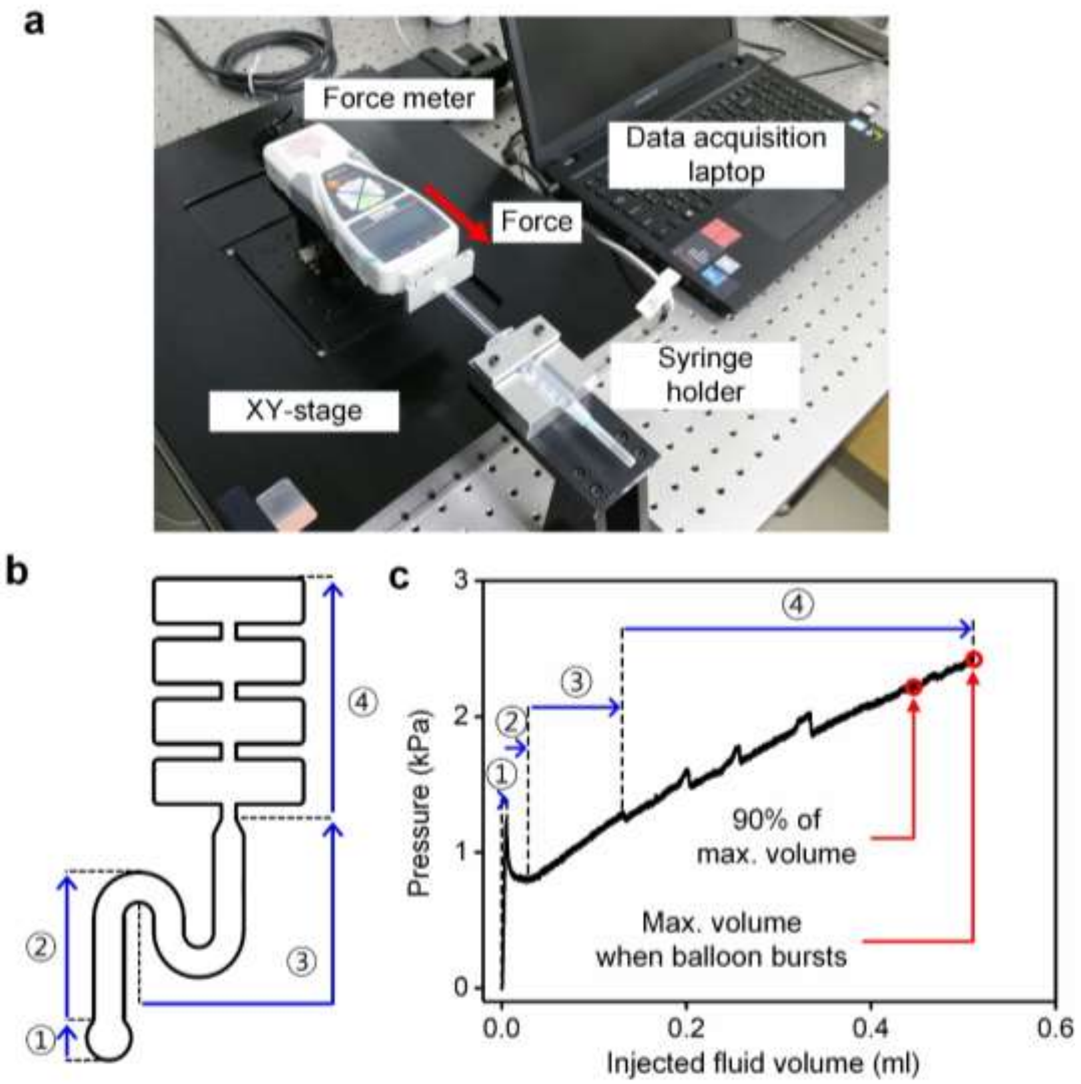

Figure S2. Establishment of a criterion for the injected fluid volume. (**a**) Setup for measurement of volume and pressure when the balloon bursts. When a force meter pushed the piston of the syringe connected to the fluidic channel of the device, a force was measured, and fluid was injected into the balloon pattern until it burst. (**b**) Balloon pattern design with the flow path of the injected fluid. (**c**) Measurement of the pressure according to the injected fluid volume. Here, the used fluid was air. A specific tendency of the measured pressure was observed according to each flow path in the balloon pattern, as shown by the blue arrows. The measured volume and pressure at bursting were 510.8 µl and 2.42 kPa, respectively. Consequently, the calculated 90% of the maximum fluid volume and pressure were 459.7 µl and 2.25 kPa, respectively.

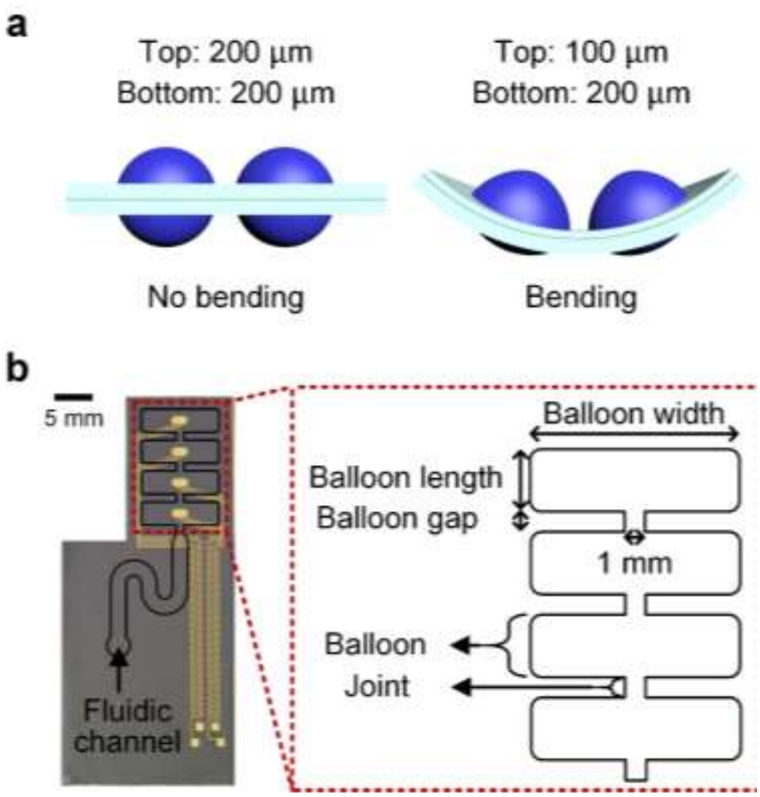

Figure S3. Two conditions to generate a bent structure. (**a**) Different thicknesses between the top and bottom PDMS layers and (**b**) a fluidic channel consisted of the balloon and joint patterns. The substrate of the device bent towards the thinner PDMS layer upon fluid injection because of the lower rigidity. In addition, as there were both balloon and joint patterns in the fluidic channel, the substrate was bent at the joints when pressure was applied to the balloons by fluid injection. To match the bent diameter of the SACE device to target nerve's diameter (2.2 mm), optimization of balloon width, length and gap was required. When the device has the bent diameter larger than nerve's diameter, all of four electrode channels cannot be contacted securely as shown in Figure 2d. Therefore, it causes low performance for signal recording because the contact pressure on nerves is not enough at the interface between electrodes and nerve surface. The larger balloon can be bent more but it is not suitable for implantation in the body, especially at the cavity around the nerve. On the other hand, the device with small balloons cannot grasp the small-sized nerves such as rabbit's sciatic nerve. First of all, joint width and balloon gap were decided as the smallest value of 1mm, because the degree of bending was dramatically deteriorated as larger joint width or balloon gap was used so that the device could not grasp the nerve. In addition, balloon size (width × length) also critically affected the degree of bending. Though larger balloon size enabled smaller bent diameter, it made balloon burst because of high pressure exerted in the balloon. Accordingly, balloon width was optimized to 10 mm as a minimum, enabling bent diameter under 2.2 mm while minimizing

the device size. Then, balloon length was optimized to 3 mm as a maximum so that adjacent balloons did not interfere each other during bending. The SACE device including balloons with 4 mm length bent less than that including balloons with 3 mm length, because balloons with 4 mm length crashed each other during the inflation and the device could not fully bend upon fluid injection.

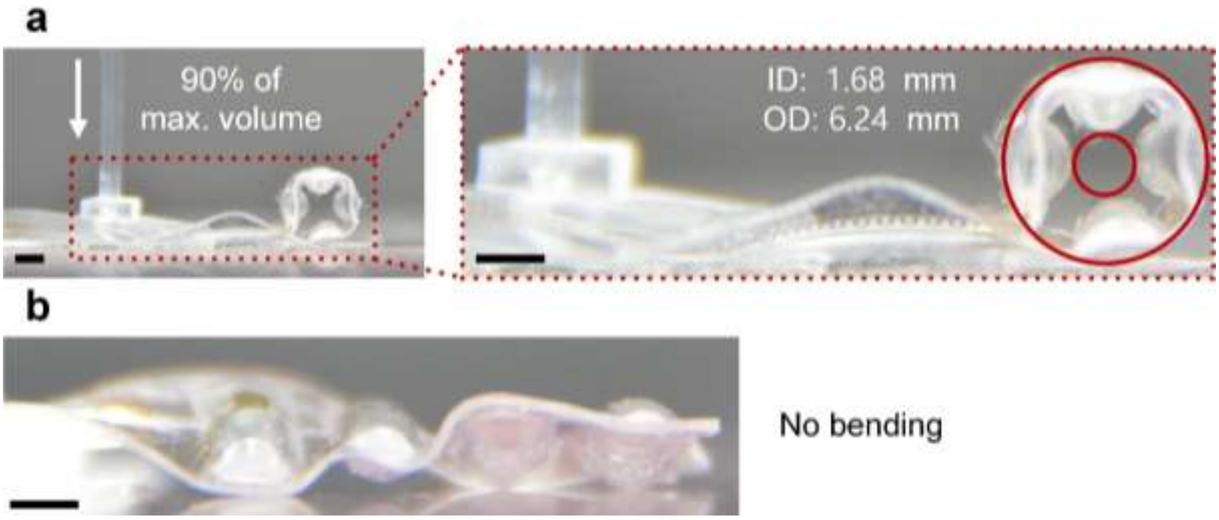

Figure S4. Effect of the thickness difference between the top and bottom PDMS layers on bending. (**a**) Different thicknesses of top and bottom PDMS layers with 100 and 200 µm, respectively, generated bending. The device with a balloon length of 3 mm and a balloon gap of 1 mm resulted in an ID of 1.68 mm and an OD of 6.24 mm. (**b**) When the same thickness of 100 µm was used for the top and bottom PDMS layers, no bending was resulted. The scale bars are 2 mm.

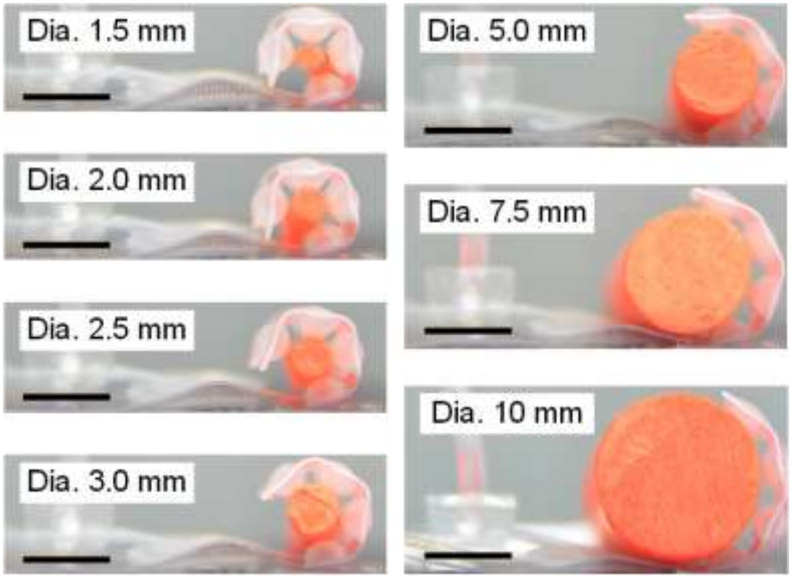

Figure S5. Demonstration of the SACE applied to nerve models with various diameters. SACE with four balloons can securely contact to nerves ranging from 1.5 to 10 mm. The scale bars are 5 mm.

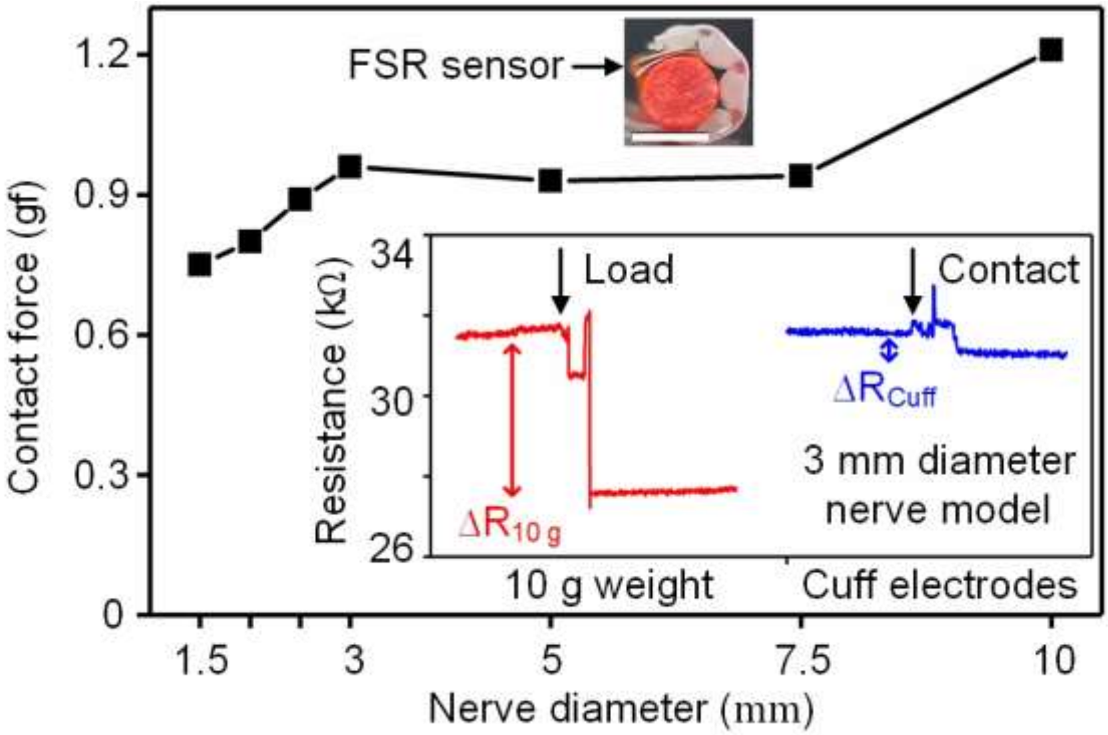

Figure S6. Measurement of contact force between electrodes and nerve model. The inset picture shows the contact force measurement from 5 mm diameter nerve model. The resistance change from the force-sensitive resistor (FSR) sensor was measured and the contact force was calculated via comparison with the resistance change when loading a 10 g weight. In the inset graphs, the 3 mm diameter nerve model was enveloped by the developed device, and the resistance changes were measured after loading a 10 g weight. The contact forces for nerve models of various sizes were calculated by comparing the resistance change when loaded with a 10 g weight. When the SACE enveloped the nerve models with diameters ranging from 1.5 to 10 mm, the contact force was measured to be from 0.75 up to 1.21 gf. The scale bar is 5 mm.

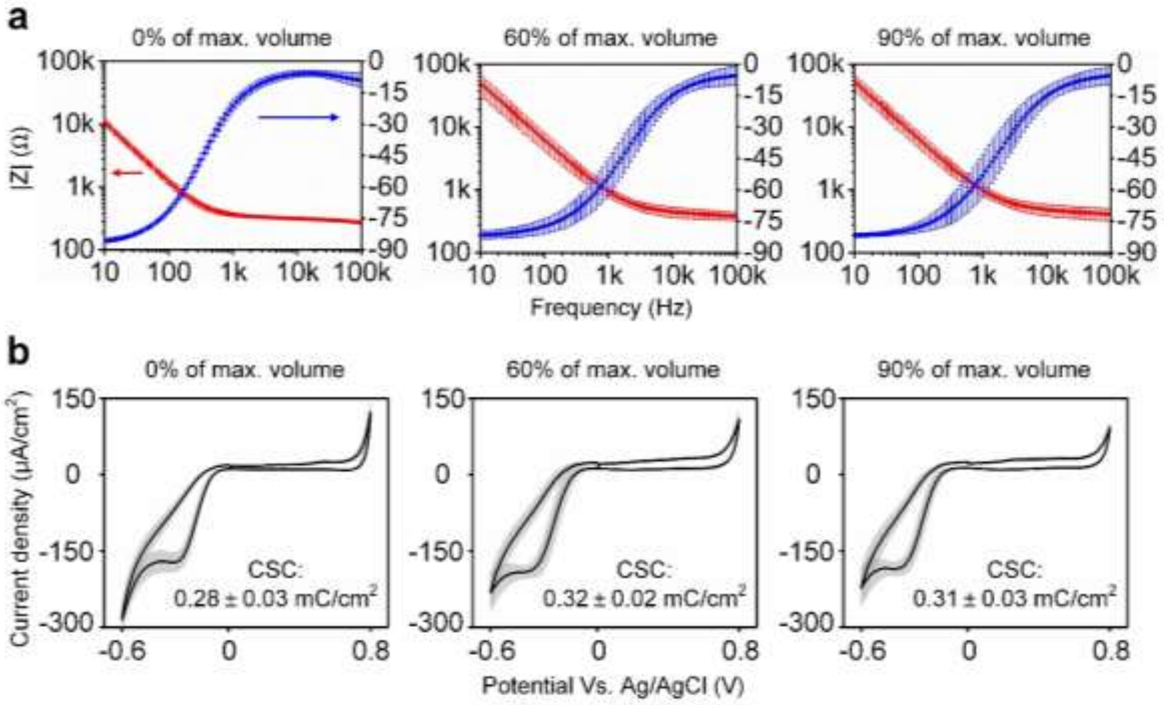

Figure S7. Electrochemical characterization depending on different injected fluid volume. (**a**) Impedance magnitude (in red) and phase (in blue) and (**b**) cyclic voltammetry (CV) curve measured from the SACE after the injection with 0, 60, and 90% of the maximum injectable fluid volume (n≥9 for each impedance and CV curve). As the injected volume increased from 0% to 90% of the maximum injectable fluid volume, the impedance magnitude at 1 kHz was observed to increase from 360 to 1000 Ω. In addition, the CSC slightly increased by 10.7% with the injected volume increase up to 90%.

a

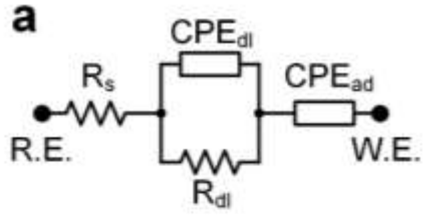

Fluid volume	Goodness of fit (x10^{-5})
0%	2.11
60%	4.62
90%	6.44

b

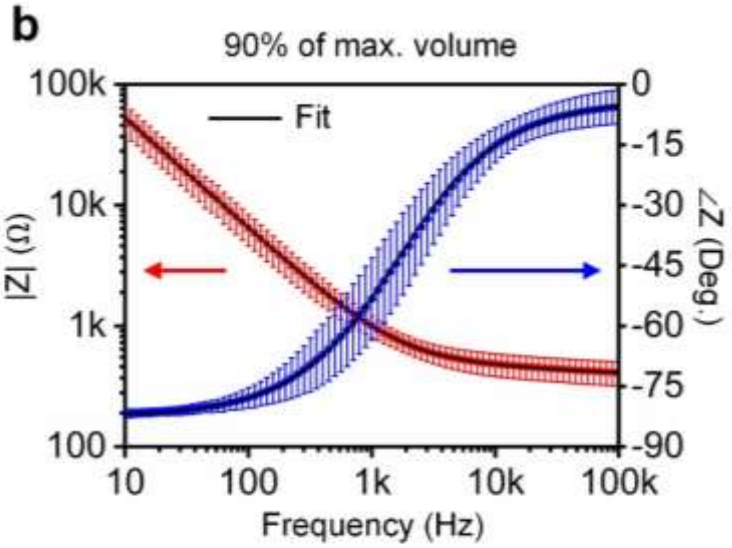

c

$$Z_{CPE} = \frac{1}{Y_{CPE}} = \frac{1}{C_{CPE}(j\omega)^{\alpha}}$$

Z : impedance (Ω) Y : admittance (S)
C : capacitance (F)
α = 0: pure resistor, α = 1: ideal capacitor

d

Fluid volume	R$_s$ (Ω)	R$_{dl}$ (Ω)	Y$_{dl}$ (μS)	α_{dl}	Y$_{ad}$ (μS)	α_{ad}
0%	42.4	302.9	4.63	0.42	1.63	0.97
60%	258.6	311.8	292	0.21	0.46	0.91
90%	322.2	333.4	257	0.26	0.42	0.92

Figure S8. Modeling of electrochemical impedance. (**a**) The measured impedance can be modeled as an adsorption equivalent circuit model with excellent goodness of fit (<10^{-4}). This model is composed of a solution resistance (R_s), a double layer resistance (R_{dl}), a double layer constant phase element (CPE_{dl}), and an adsorption constant phase element (CPE_{ad}). (**b**) From the impedance magnitude (in red) and phase (in blue), the black line shows a perfect fit of the adsorption model with the measurement. (**c**) Impedance of CPE (Z_{CPE}) can be expressed in terms of capacitance (C_{CPE}) and alpha (α) that has a value between 0 to 1. This indicates whether the CPE is close to the resistor or capacitor. (**d**) The values of R_s, R_{dl}, CPE_{dl}, and CPE_{ad} in the table were calculated according to the equivalent circuit.

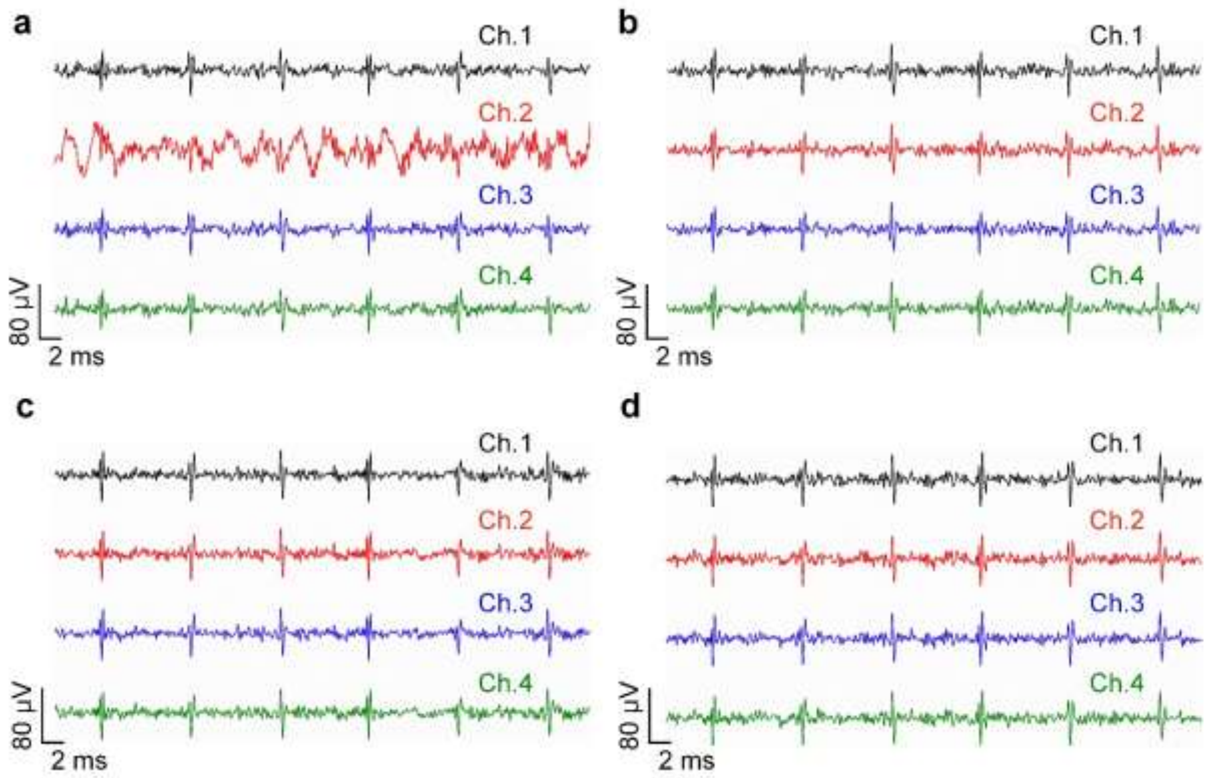

Figure S9. Action potentials (APs) recorded from the agarose nerve model. APs from four electrode channels (black, red, blue, and green lines) were recorded after injection with (**a**) 60%, (**b**) 70%, (**c**) 80%, and (**d**) 90% of the maximum fluid volume. The recorded AP from electrode channel 2 after injection with 60% of the maximum fluid volume contained significant noise since the electrodes had poor contact with the nerve model. This result was caused by the bent device's relatively large ID (2.05 mm) compared to the nerve model's diameter (2 mm). In contrast, when 90% of the maximum fluid volume was injected, the device enveloped the nerve model with firm contact at all four electrodes.

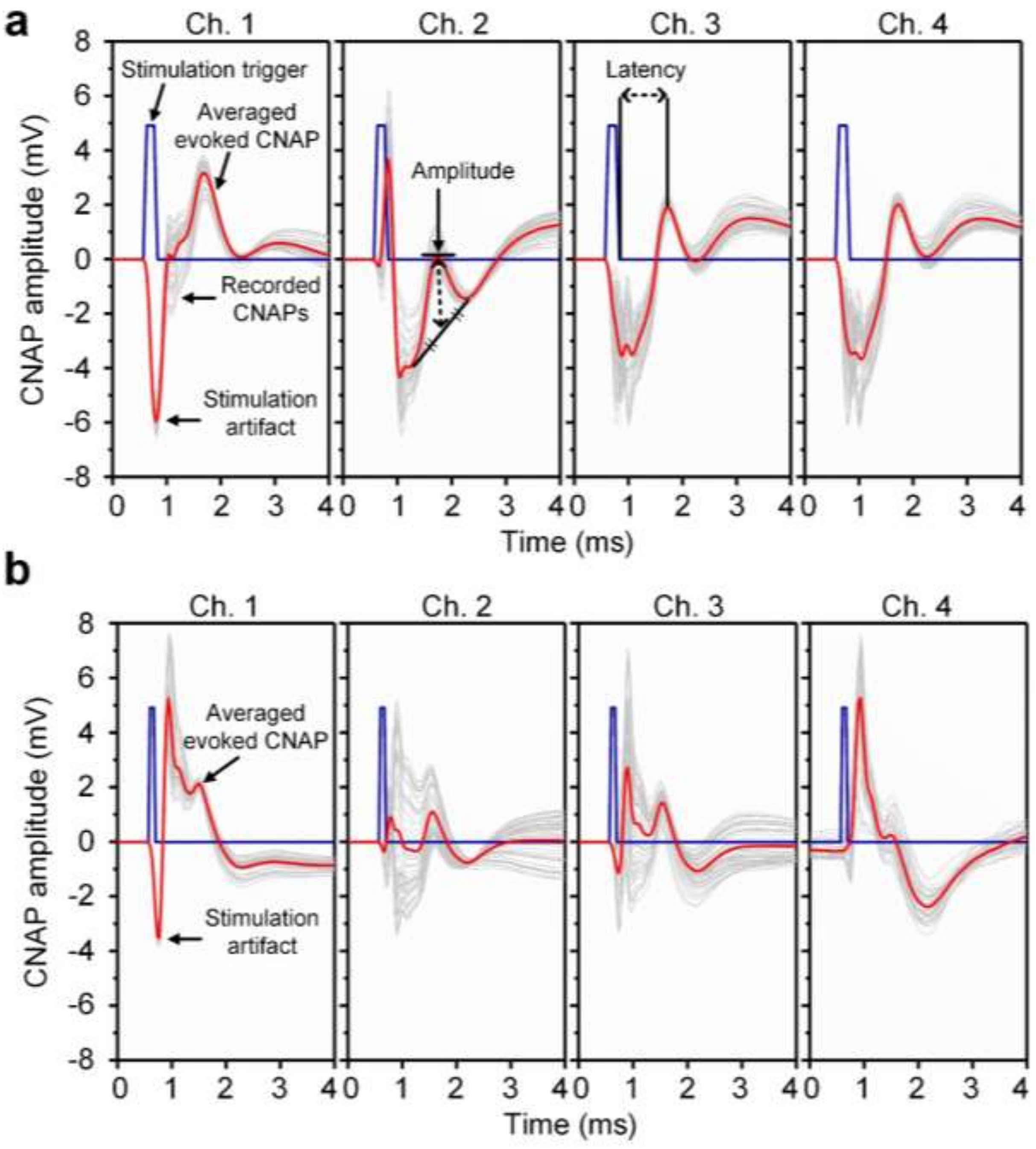

Figure S10. CNAP recordings using the SACE. CNAP responses (gray) recorded from the four electrode channels following stimulation with pulse widths of (**a**) 100 μs and (**b**) 50 μs ($n \geq 50$). The red and blue lines indicate the averaged CNAP and stimulation trigger, respectively. The stimulation trigger, expressed by a 5 V DC output, presents synchronized timing with a biphasic pulse for stimulation. After the stimulation, a stimulation artifact and an evoked CNAP were observed.

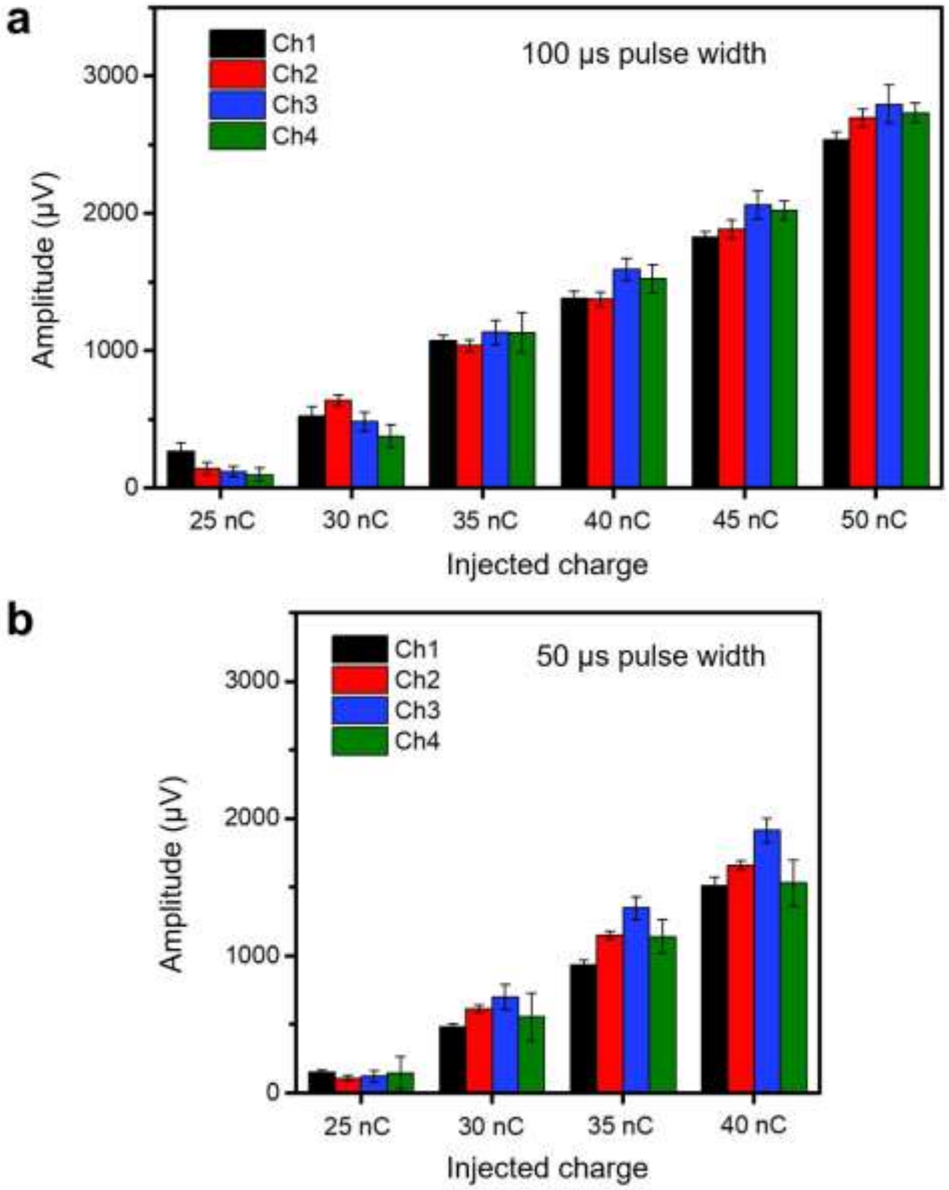

Figure S11. CNAP amplitude changes depending on injected charge by different stimulation intensity. CNAP responses to the stimulation of rabbit sciatic nerve with (**a**) 100 us and (**b**) 50 us in pulse width were acquired from four channels of cuff electrodes ($n \geq 50$). From both results, the pressure exerted by four protrusions of the SACE device on the nerve was slightly different, but the difference was not significant. The differences in amplitude by four channels were not so significant that all four channels could detect different amplitudes depending on different injected charges. Consequently, the amplitude of CNAP clearly increased upon the increased injected charge. In addition, injected charge over 25 nC could activate the rabbit sciatic nerve. The black-, red-, blue-, and green-colored lines indicate the recorded CNAP amplitudes from electrode channel 1 to 4, respectively.

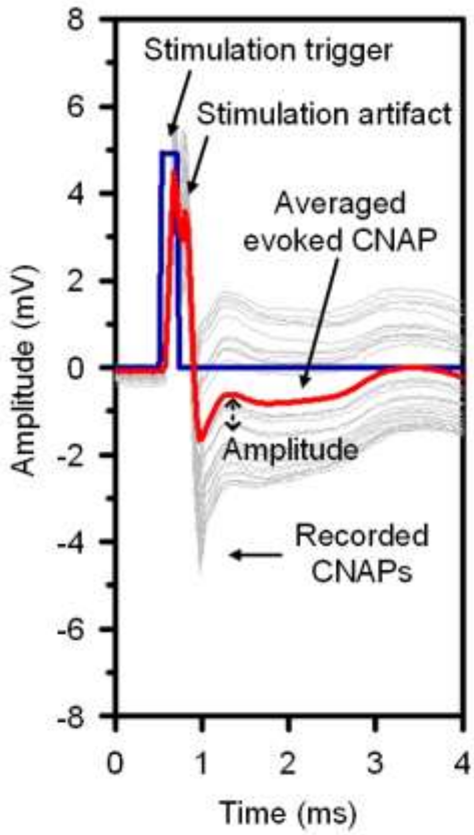

Figure S12. CNAP response recorded in the experimental configuration with a 20 mm distance between the stimulation and recording electrodes. The nerve was stimulated by a hook electrode using a biphasic pulse of 0.3 mA, 100 µs, and 1 Hz. Then, CNAP responses was recorded by the SACE (*n* = 35). The evoked CNAP had an amplitude of 0.59 mV and a conduction speed of 39.2 ms. The gray-, red-, and blue-colored lines indicate the recorded CNAPs, averaged CNAP, and stimulation trigger, respectively.

Non-penetrating type cuff electrodes	SNR (a.u.)	Noise level (μV)	Duration (week)
SACE device	13.2	16	5
ref 1	5.2	25	Acute
ref 2	3	10	6
ref 3	4.7	40	12
ref 4	6.4	17	2
ref 5	1.5	20	3
ref 6	10	86	4
ref 7	6.5	5	24

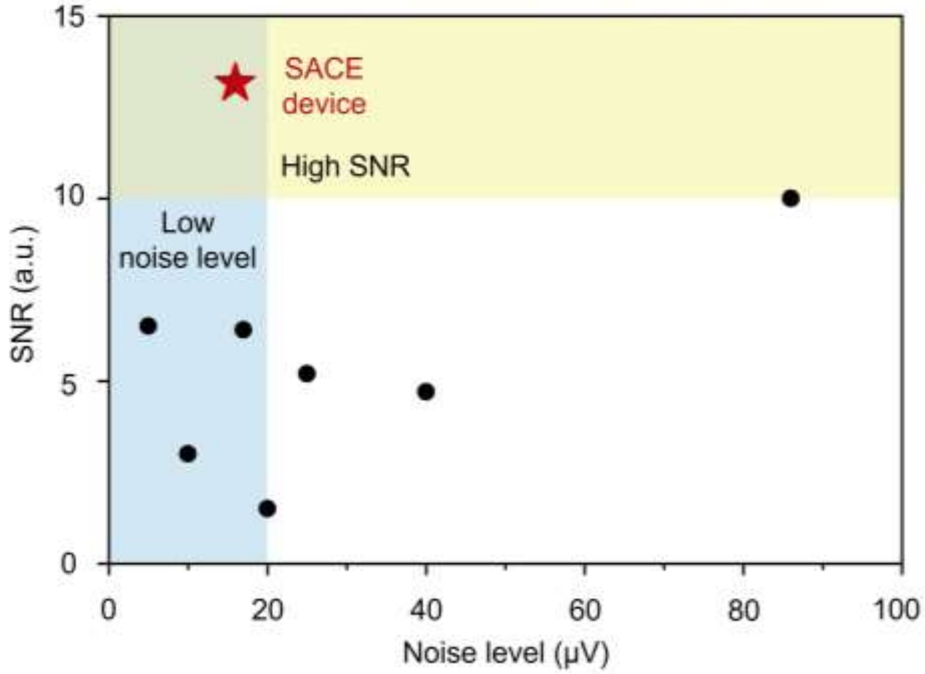

Figure S13. Superiority of the SACE device. The SACE device shows the highest SNR of 13.2 and relatively low noise level of 16 μV, compared to previous cuff electrodes.[1–7]

1. Y. C. Zhang, N. Zheng, Y. Cao, F. L. Wang, P. Wang, Y. J. Ma, B. W. Lu, G. H. Hou, Z. Z. Fang, Z. W. Liang, M. K. Yue, Y. Li, Y. Chen, J. Fu, J. Wu, T. Xie, X. Feng, *Science Advances* **2019**, 5.
2. K. I. Song, H. Seo, D. Seong, S. Kim, K. J. Yu, Y. C. Kim, J. Kim, S. J. Kwon, H. S. Han, I. Youn, H. Lee, D. Son, *Nature Communications* **2020**, 11.
3. D. N. Heo, H. J. Kim, Y. J. Lee, M. Heo, S. J. Lee, D. Lee, S. H. Do, S. H. Lee, I. K. Kwon, *Acs Nano* **2017**, 11, 2961.
4. R. Y. Tang, C. L. Zhang, B. X. Liu, C. Y. Jiang, L. Wang, X. R. Zhang, Q. Huang, J. Liu, L. Li, *Biosensors & Bioelectronics* **2022**, 216.
5. J. Jeong, T. H. Kim, S. Park, J. Lee, U. Chae, J. Y. Jeong, S. Park, S. Kim, I. J. Cho, Y. Jung, H. Yi, *Chemical Engineering Journal* **2023**, 465.
6. T. M. Otchy, C. Michas, B. Lee, K. Gopalan, V. Nerurkar, J. Gleick, D. Semu, L. Darkwa, B. J. Holinski, D. J. Chew, A. E. White, T. J. Gardner, *Nature Communications* **2020**, 11.
7. C. Delianides, D. Tyler, G. Pinault, R. Ansari, R. Triolo, *Neuromodulation* **2020**, 23, 754.

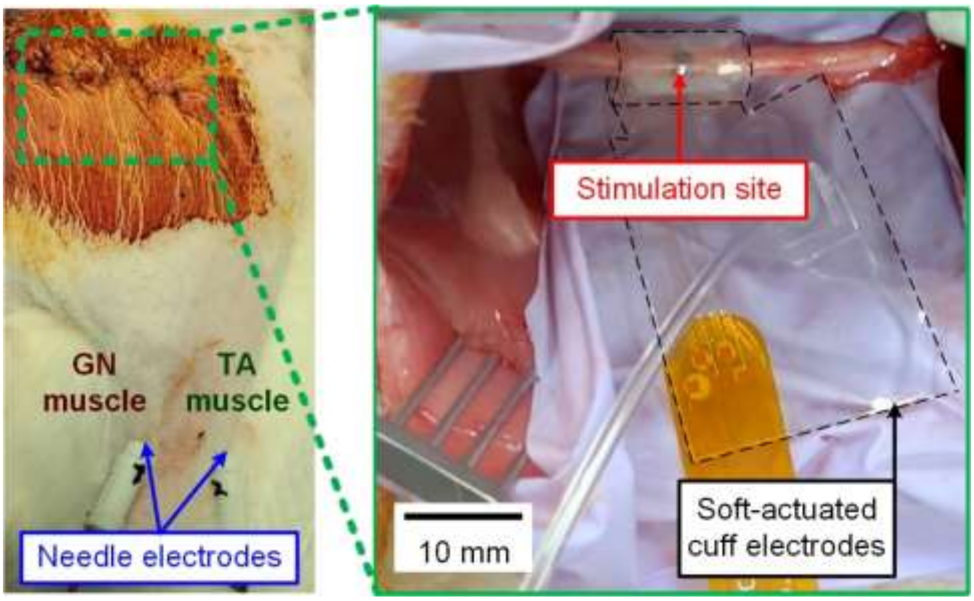

Figure S14. Experimental setup for nerve stimulation. Configuration of the SACE for stimulation and the needle-type electrodes for EMG recording from the GN and TA muscles. The developed device enveloped the sciatic nerve, while the needle electrodes were inserted into the GN and TA muscles. The GN muscle site was relatively closer to the stimulation site than the TA muscle site.

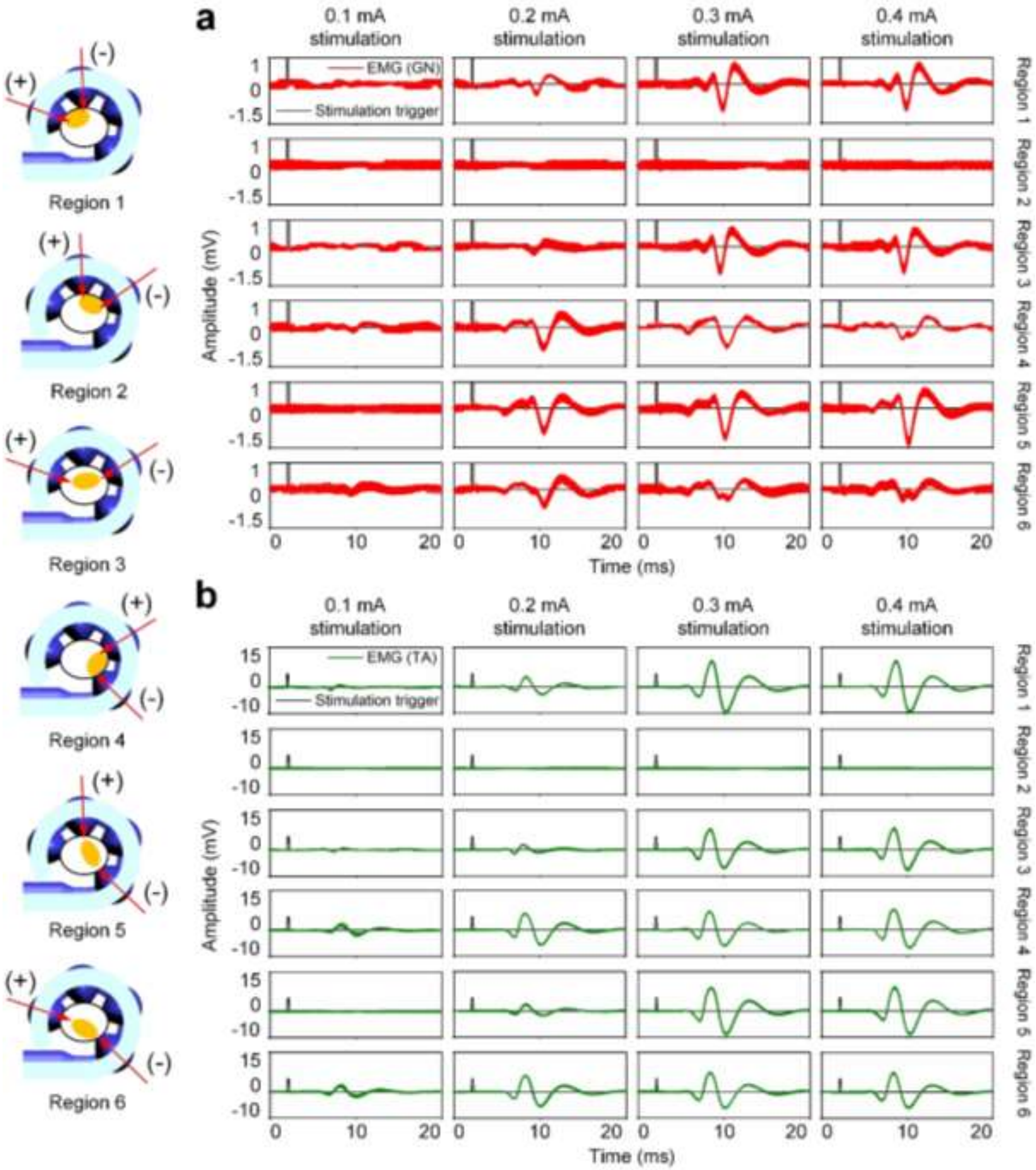

Figure S15. EMG responses to nerve stimulation up to 0.4 mA amplitude. EMG responses recorded from the (**a**) GN muscle (red line) and (**b**) TA muscle (green line) upon stimulation (black line) with amplitudes of 0.1, 0.2, 0.3, and 0.4 mA (n = 20). Both GN and TA muscles were activated by electrical stimulation at all regions except for region 2, indicating selective stimulation.

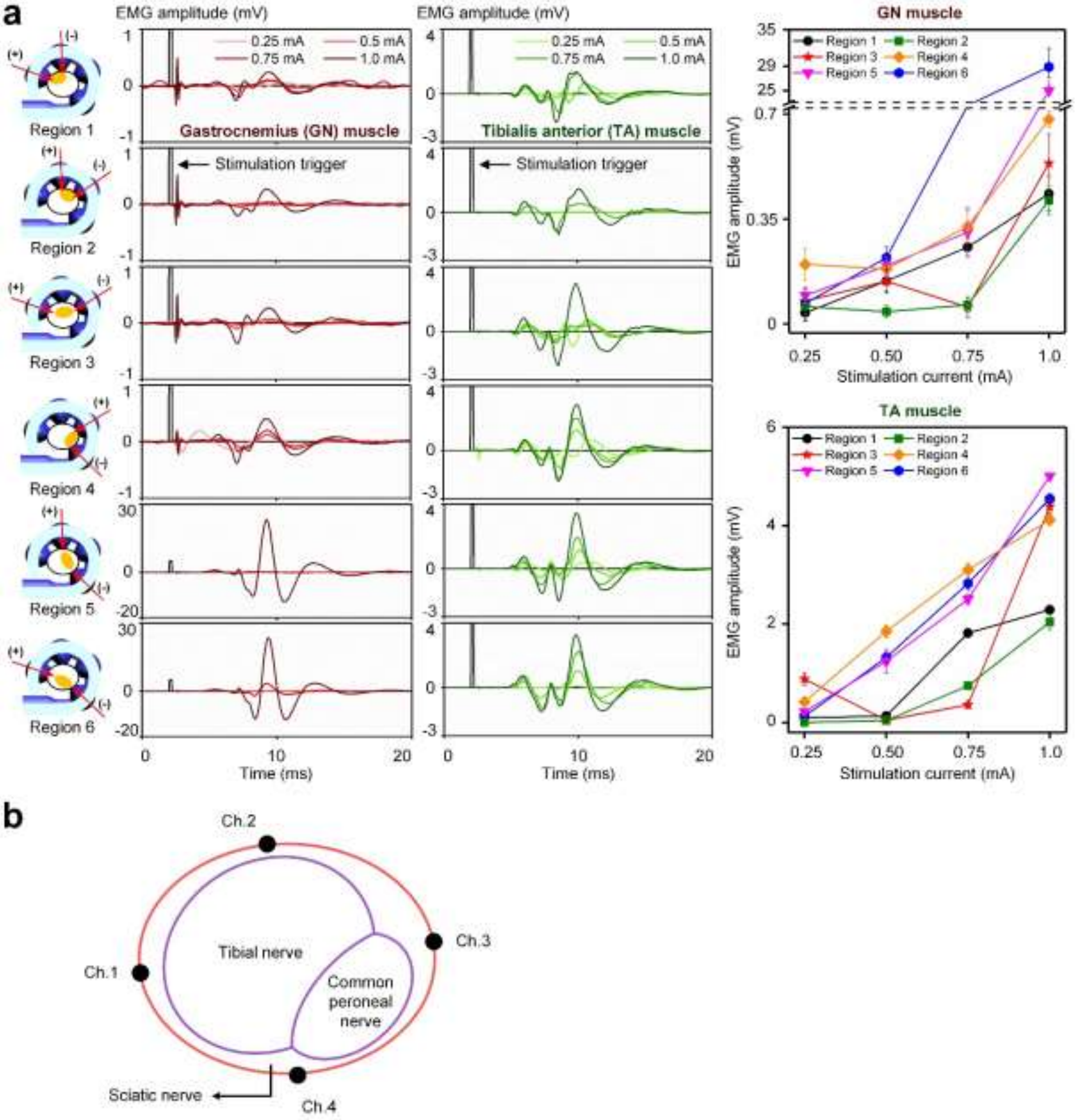

Figure S16. EMG responses of the GN and TA muscles to nerve stimulation, according to six stimulation regions with amplitudes of 0.25, 0.5, 0.75, and 1 mA (*n* ≥ 30). And presumed positions of tibial and common peroneal nerves inside the sciatic nerve according to spatial information of four stimulation channels. (**a**) For the GN muscle, the stimulations with a 1 mA amplitude at regions 5 and 6 strongly activated the GN muscle with evoked EMG signals over 25 mV. In particular, stimulation of region 6 resulted in the strongest activation of the GN muscle. On the other hand, stimulation of region 2 resulted in almost no activation of the GN muscle. For the TA muscle, stimulation with 1 mA amplitude at regions 3, 4, 5, and 6 induced the fully activated TA muscle. However, stimulation of region 3 showed relatively deteriorated performance for TA muscle activation, with evoked EMG response of 0.9 mV after stimulation with 0.75 mA. Stimulation of region 4 resulted in the strongest

activation of the TA muscle, with a large EMG amplitude of 1 mV after stimulation with 0.25 mA. On the other hand, stimulation of region 2 started to generate EMG responses upon stimulation with an amplitude over 0.75 mA, presenting the worst performance for TA muscle activation. Consequently, stimulation of regions 6 and 4 showed the most effective activation of the GN and TA muscles, respectively. (**b**) From spatial information of GN and TA muscle activation, effective and non-effective regions for each GN or TA muscle could be identified. The tibial and common peroneal nerves might be located near channel 1 to 4 (region 6) and channel 3 to 4 (region 4), respectively, and both should be far from channel 2 to 3 (region 2). Consequently, we could suppose the positions of tibial and common peroneal nerves inside the sciatic nerve.

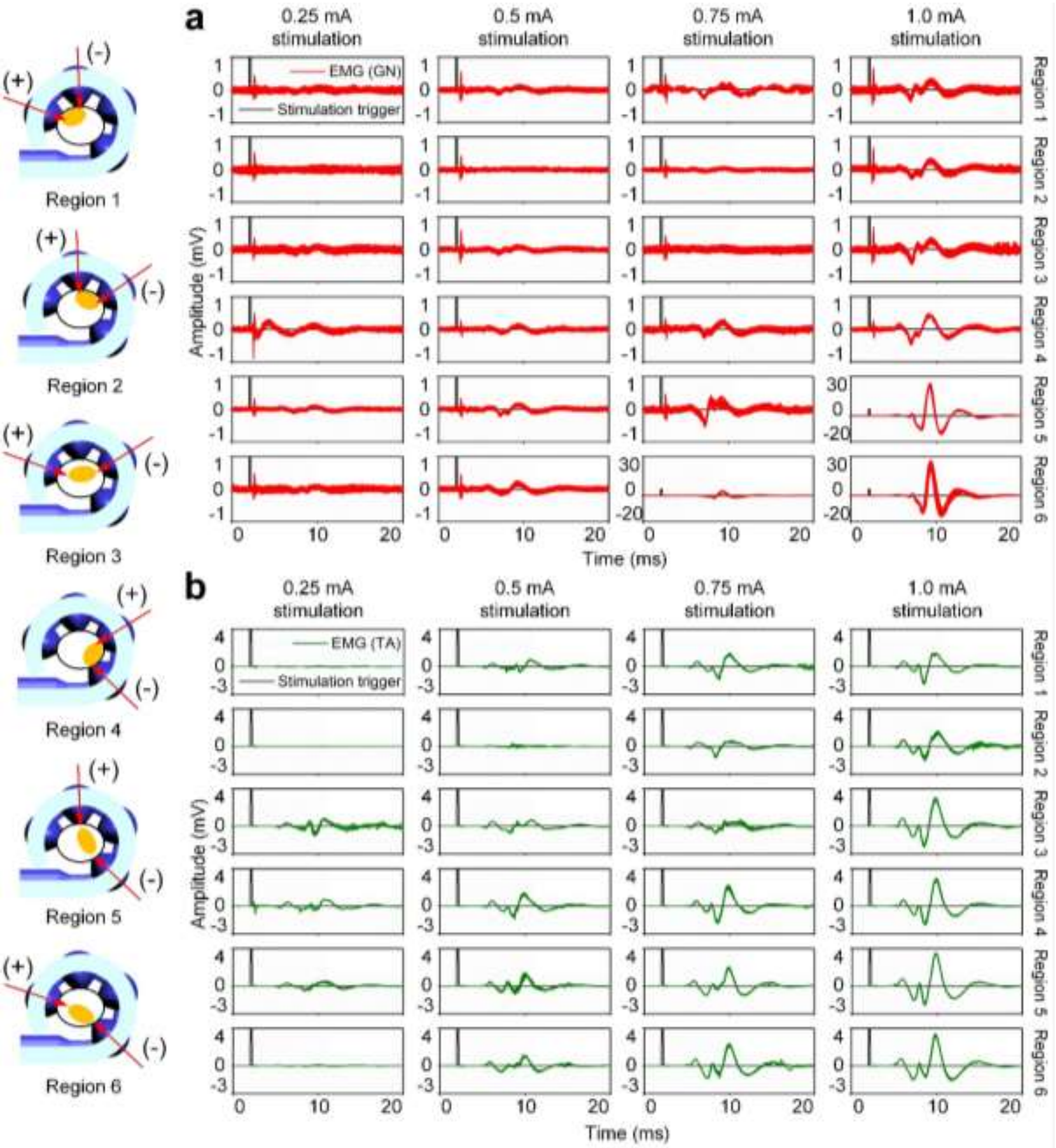

Figure S17. EMG responses to nerve stimulation up to 1 mA amplitude. EMG responses recorded from the (**a**) GN muscle (red line) and (**b**) TA muscle (green line) upon stimulation (black line) with amplitudes of 0.25, 0.5, 0.75, and 1 mA ($n \geq 30$). The GN muscle was highly activated at the regions 5 and 6. On the other hand, smaller EMG responses were observed when the other four regions 1 to 4 were stimulated. The TA muscle was relatively more activated when regions 3 to 6 were stimulated. In contrast, nerve stimulation at regions 1 and 2 generated relatively low EMG responses. The amplitudes of the EMG response differed considerably between highly activated and less activated GN muscle, while the EMG amplitudes were similar in the case of TA muscle.

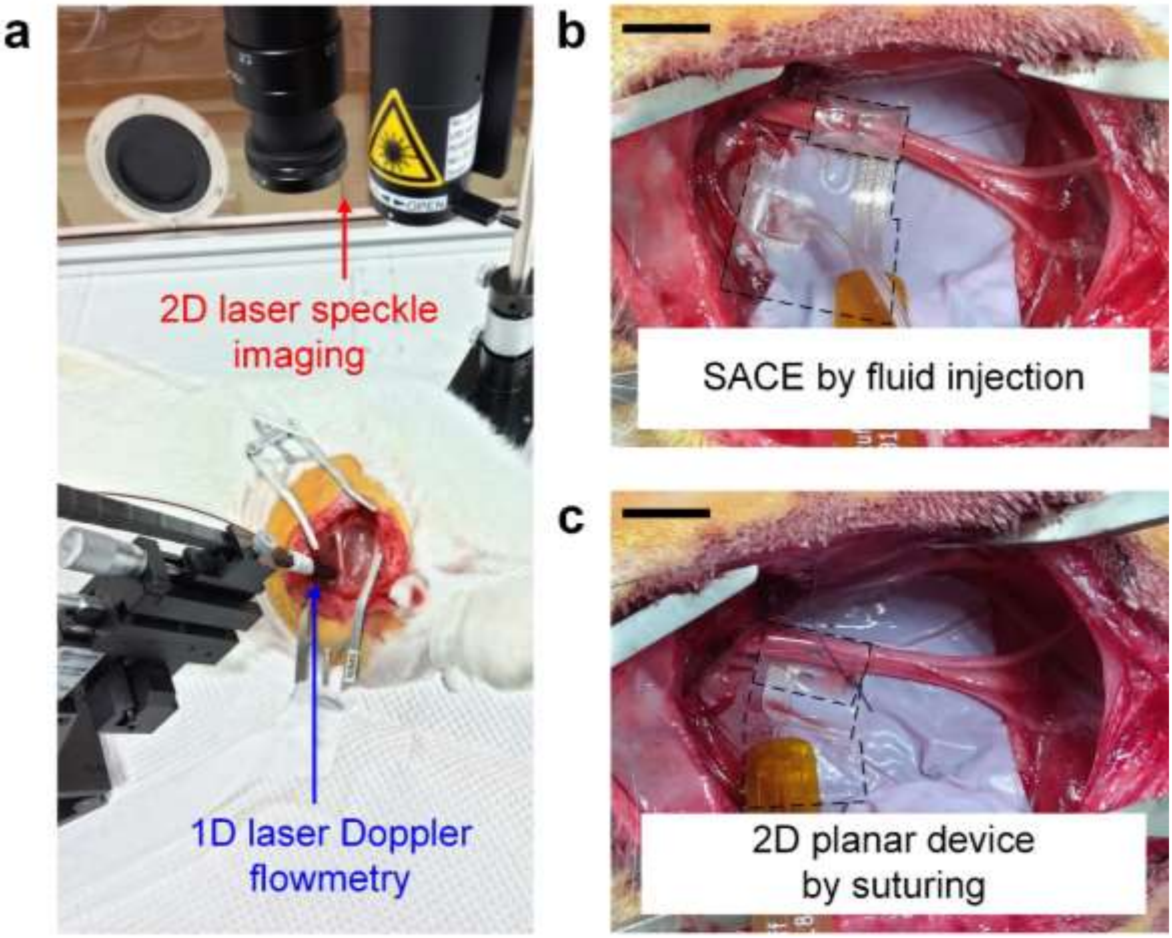

Figure S18. Setup for measurement of the blood flow at the nerve surface. (**a**) 2D laser speckle imaging and 1D laser Doppler flowmetry for acquisition of blood flow in the speckle image and blood flow index, respectively. For evaluation of the contact pressure between electrodes and the nerve, (**b**) the SACE were fixed by fluid injection, whereas (**c**) the 2D planar devices were implanted on the nerve by suturing, which is a conventional procedure to apply electrodes at a nerve. The scale bars are 10 mm.

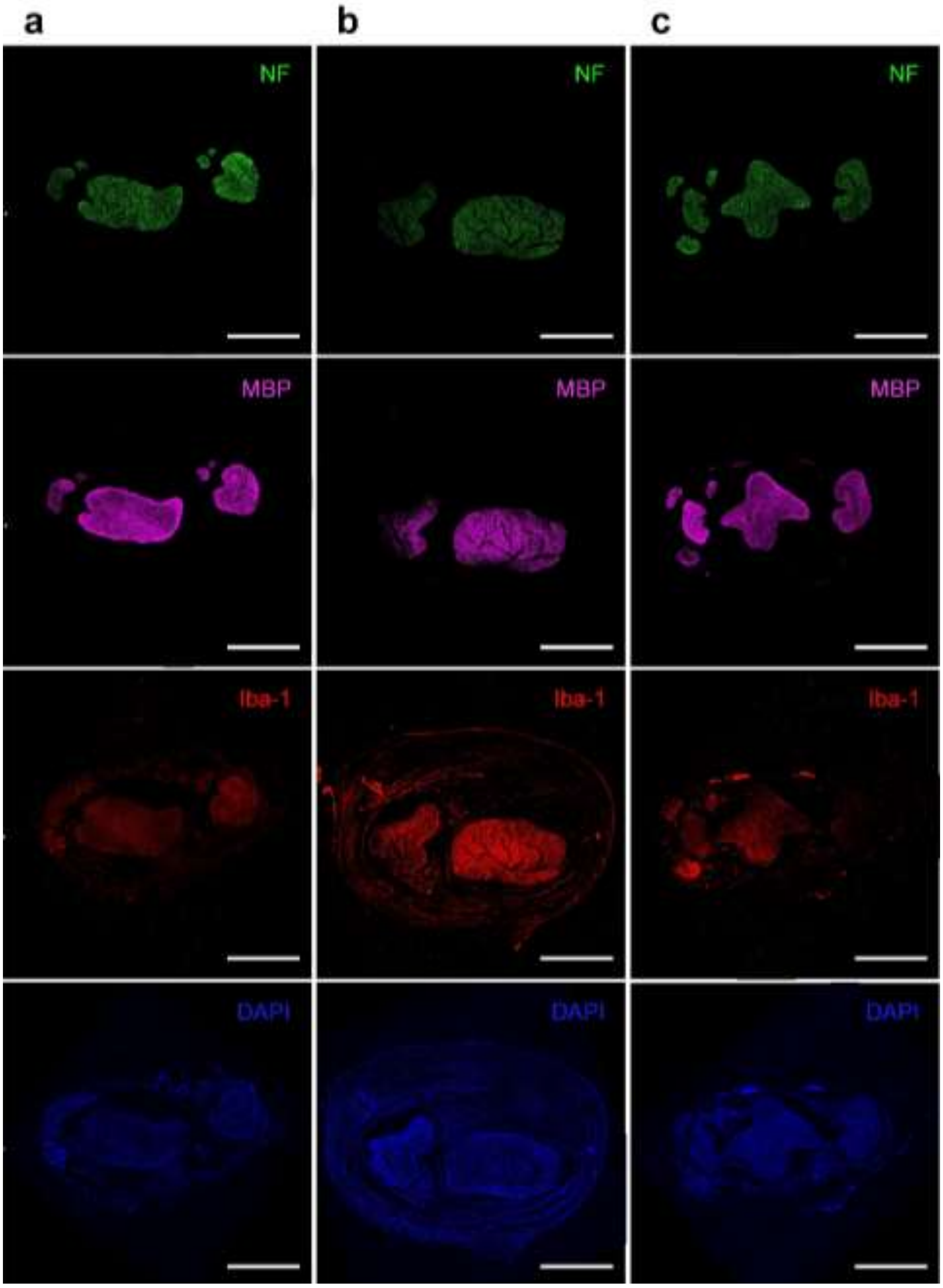

Figure S19. Fluorescent images from IHC staining of the sciatic nerve. Three groups of nerve samples were used: (**a**) the control, (**b**) the nerve after 2 weeks of device implantation, and (**c**) the nerve after 12 weeks of device implantation. Green and magenta fluorescence represent neurofilament (NF) and myelin (maltose binding protein, MBP), indicating the healthy nerve. On the other hand, red fluorescence represents macrophage (Iba-1), exhibiting an inflammatory activity. Blue fluorescence represents nuclei of cells through a counter staining. The scale bars are 500 μm.

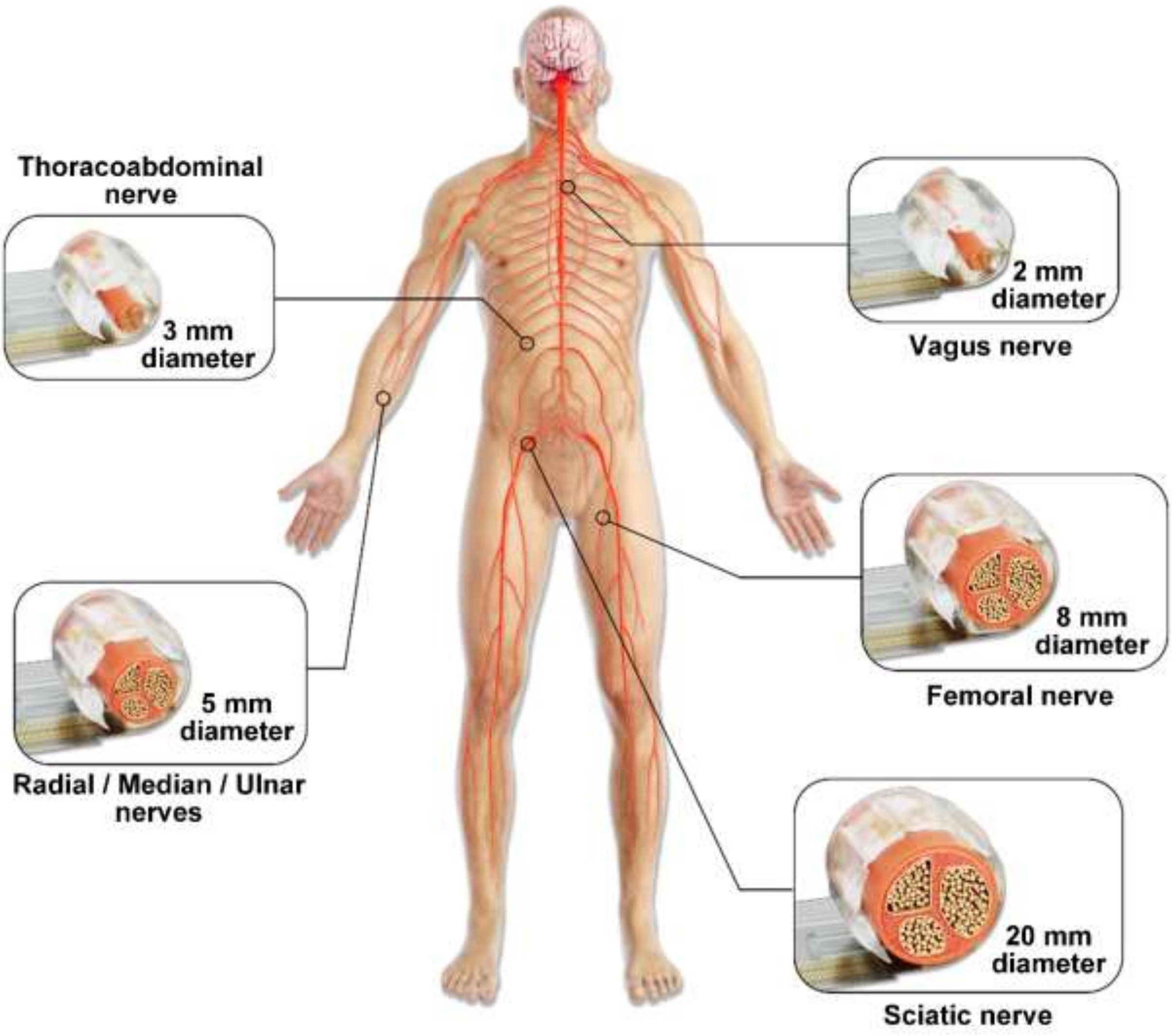

Figure S20. Potential applications of the SACE for human. Schematic illustration of the nerves of various sizes where the SACE can be applied in the human nervous system, such as the vagus (diameter: ~2 mm), thoracoabdominal (~3 mm), radial/median/ulnar (~5 mm), femoral (~8 mm), and sciatic (~20 mm) nerves. Bent diameters of the developed device can vary depending on different design and number of balloons.

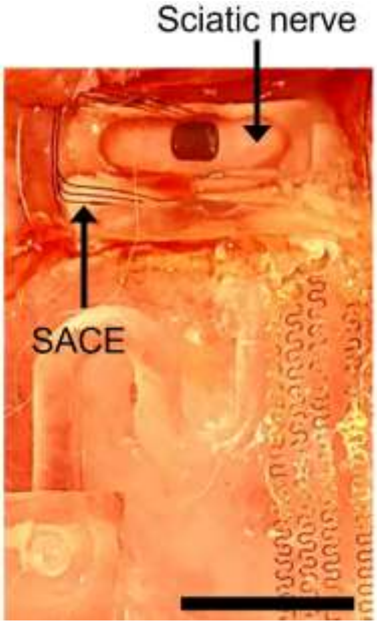

Figure S21. Image of the implanted SACE device on a rabbit's sciatic nerve after 7 months of implantation. There was no significant inflammation around the nerve contacted with the device, implicating the biocompatibility and stability of the SACE over long-term. The scale bar is 5 mm.

Table. S1. Six types of devices with different dimensions using four design parameters. Figure S4a presents the optimized conditions for the smallest bending diameter. On the other hand, figure S4b corresponds to thickness changes in the top and bottom PDMS layers. Figure 1e–h corresponds to balloon dimension changes of the fluidic channel.

Sample type	Top thickness (μm)	Bottom thickness (μm)	Balloon length (mm)	Balloon gap (mm)
Figure S4a	100	200	3	1
Figure S4b	200	200	3	1
Figure 1e	200	400	3	1
Figure 1f	100	200	1	1
Figure 1g	100	200	2	1
Figure 1h	100	200	3	2

Supporting Videos

Video S1. Cyclic bending of the SACE.

Video S2. Enveloping a nerve model with a diameter of 2 mm.

Video S3. No signals recorded by swaying of the leg.

Video S4. CNAP recordings by voluntary leg movement.

Video S5. CNAP recordings by extension and flexion in short period.

Video S6. CNAP recordings by extension and flexion in long period.

Video S7. Twitching of rabbit leg upon different amplitudes of stimulation.

Video S8. Selective GN muscle stimulations.

Video S9. Selective TA muscle stimulations.

Video S10. Both GN and TA muscles stimulations.
